# Microinjection of CART peptide into the nucleus accumbens medial shell attenuates methamphetamine-induced anxiety-like behaviors via restoration of GABA_B_ receptor membrane expression

**DOI:** 10.1038/s41598-026-46389-x

**Published:** 2026-03-29

**Authors:** Huiying Zhang, Zhuoxuan Yu, Qiang Fu, Jianhua Yang, Mingzhu Yan, Zhenzhen Hu

**Affiliations:** 1 Department of Pathophysiology, School of Basic Medical Sciences, Jiangxi Medical College, Nanchang University, Nanchang, 330031 Jiangxi China; 2 The First Clinical Medical College, Jiangxi Medical College, Nanchang University, Nanchang, 330006 Jiangxi China; 3 Department of Respiration, Department Two, Jiangxi Provincial People’s Hospital, Nanchang, 330006 Jiangxi China; 4 Department of Physiology, School of Basic Medicine, Jiangxi Medical College, Nanchang University, Nanchang, 330031 Jiangxi China; 5 Huankui Academy, Jiangxi Medical College, Nanchang University, Nanchang, 330031 Jiangxi China; 6 Jiangxi Provincial Key Laboratory of Brain Science and Brain Health, School of Basic Medical Sciences and Institute of Biomedical Innovation, Jiangxi Medical College, Nanchang University, Nanchang, 330031 Jiangxi China

**Keywords:** Methamphetamine, Anxiety, Nucleus accumbens, Cocaine- and amphetamine-regulated transcript peptide, Gamma aminobutyric acid type B receptor, Drug discovery, Neuroscience

## Abstract

Methamphetamine (METH) abuse often leads to anxiety-like behaviors, and the cocaine- and amphetamine-regulated transcript (CART) peptide, which is abundant in the nucleus accumbens (NAc), has shown promise in mitigating behavioral effects of psychostimulants. However, the role of CART peptide in the NAc medial shell in METH-induced anxiety remains poorly understood. In this study, following METH withdrawal and reinstatement, rats exhibited significant anxiety-like behaviors. Immunofluorescence analysis revealed a substantial increase in the proportion of c-Fos⁺/NeuN⁺ and CART⁺/NeuN⁺ cells within the NAc medial shell. Western blot and immunofluorescence consistently showed upregulated CART peptide expression and reduced gamma aminobutyric acid type B receptor (GABA_B_R) levels in this region. Further immunofluorescence staining confirmed decreased GABA_B_R expression specifically within CART-positive neurons. Microinjection of CART peptide into the NAc medial shell attenuated METH-induced anxiety-like behaviors, normalized the hyperactivity of neurons, and restored GABA_B_R expression to baseline levels. Molecular docking and co-immunoprecipitation suggest a potential interaction between CART and GABA_B_R. These protective effects were abolished by the GABA_B_R antagonist CGP55845. Overall, our findings demonstrate that CART peptide delivery into the NAc medial shell alleviates METH-induced anxiety-like behaviors by rescuing GABA_B_R membrane expression, highlighting a potential therapeutic pathway for METH-related anxiety.

**Supplementary Information:**

The online version contains supplementary material available at 10.1038/s41598-026-46389-x.

## Introduction

Methamphetamine (METH) is a widely abused synthetic psychostimulant, with an estimated 34.2 million users worldwide between the ages of 15 and 64^1^. Among adolescents aged 12 to 15, approximately 4.05% are affected by METH abuse^2^. Chronic METH use frequently leads to addiction, commonly manifested as compulsive drug-seeking behavior and comorbid psychiatric symptoms such as anxiety^3,4^. Despite recent research proposing potential intervention strategies such as gut microbiota and exosome modulation^5–7^, effective clinical interventions for METH addiction remain limited^8^, underscoring the urgent need to identify novel therapeutic targets for both addiction and METH-induced anxiety.

The cocaine- and amphetamine-regulated transcript (CART) peptide, a highly conserved neuropeptide, is abundantly expressed in brain regions including the nucleus accumbens (NAc), medial prefrontal cortex (mPFC), and ventral tegmental area. It plays important roles in appetite regulation, energy homeostasis, and behaviors related to drug addiction^9^. Within the NAc, CART peptide is predominantly expressed in GABAergic medium spiny neurons (MSNs)^10–12^ and has attracted considerable research interest. For instance, cocaine administration significantly increases the proportion of cells co-expressing CART and c-Fos in the NAc, suggesting drug-induced activation of CART-positive neurons^13^. Additionally, emerging evidence links CART signaling to anxiety regulation. Human studies indicate that missense mutations in the *cartpt* gene are associated with elevated anxiety^14^, and rodent models further support a role for CART in anxiety-like behaviors^15,16^. Microinjection of CART peptide (1 µg/side) into the NAc significantly attenuates both cocaine- and METH-induced hyperlocomotion^17,18^. Nevertheless, the potential role and mechanisms of exogenous CART peptide in modulating METH-induced anxiety-like behaviors within the NAc remain poorly understood.

The gamma aminobutyric acid type B receptor (GABA_B_R), a class C G protein-coupled receptor (GPCR), functions as an obligate heterodimer composed of GABA_B1_ and GABA_B2_ subunits. These subunits dimerize via coiled-coil interactions within their C-terminal domains, facilitating surface membrane expression by masking endoplasmic reticulum retention signals present in GABA_B1_ subunit^19–21^. GABA_B_R has been implicated in several neuropsychiatric disorders, including anxiety, depression, and substance use disorders^22,23^. For example, in the mPFC—an upstream region of the NAc—Notch1 signaling downregulates GABA_B1_R expression via the transcriptional repressor Hes1, contributing to METH-induced behavioral sensitization^24^. Furthermore, microinjection of the GABA_B_R agonist baclofen into the NAc medial shell reduces anxiety-like behaviors in the elevated plus maze (EPM)^25^, and oral administration of the positive allosteric modulator GS39783 decreases anxiety-like phenotypes in both the EPM and elevated zero maze^26^. Despite these advances, it remains unclear whether GABA_B_R contributes to CART peptide-mediated alleviation of METH-induced anxiety in the NAc.

In this study, we employed western blotting, open field, and elevated plus maze tests to demonstrate that microinjection of CART peptide into the NAc medial shell mitigates METH-induced behavioral sensitization and anxiety-like behaviors. We also observed that CART peptide delivery into the NAc medial shell normalizes METH-evoked increases in c-Fos and CART expression and restores GABA_B_R membrane levels in CART-positive neurons. Molecular docking and co-immunoprecipitation (Co-IP) suggest a potential interaction between CART and GABA_B_R. Critically, the protective behavioral effects of CART were reversed by the GABA_B_R antagonist CGP55845, supporting the conclusion that GABA_B_R mediates the anxiolytic actions of CART peptide in the context of METH exposure.

## Results

### METH induces behavioral sensitization and anxiety-like behaviors in rats

**Fig. 1:**
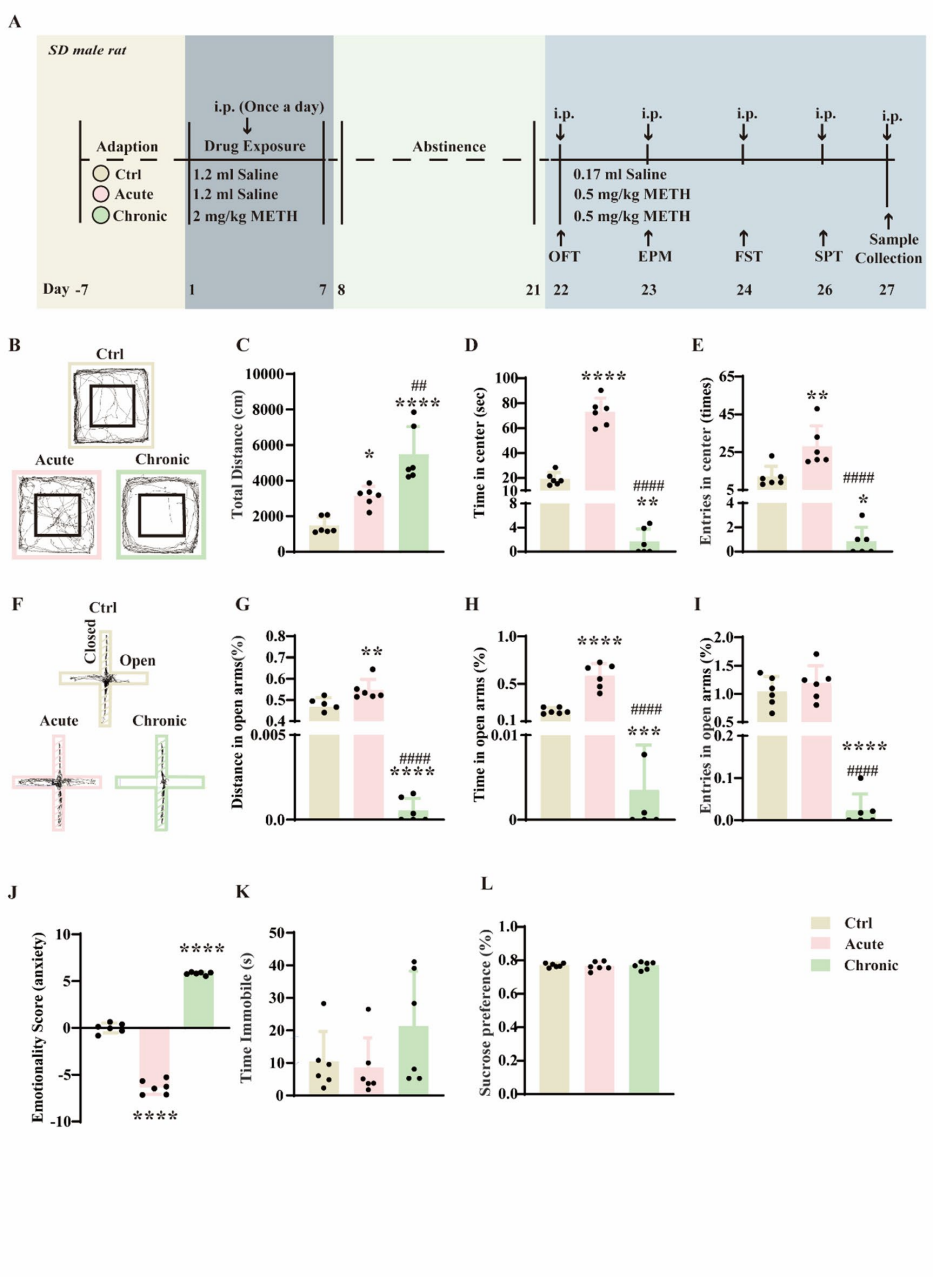
Acute methamphetamine (METH) administration reduces baseline anxiety in rats, whereas chronic METH exposure induces behavioral sensitization and promotes anxiety-like behaviors. (**A**) Experimental timeline for acute and chronic METH treatments in rats. (**B**) Representative movement traces of rats in the open field test (OFT) (*n* = 6). (**C**) Total distance travelled in the OFT (*n* = 6). (**D**) Time spent in the center zone of the OFT (*n* = 6). (**E**) Number of entries into the center zone of the OFT (*n* = 6). (**F**) Representative movement traces in the elevated plus maze (EPM). (**G**) Percentage of distance travelled in the open arms of the EPM (*n* = 6). (**H**) Percentage of time spent in the open arms of the EPM (*n* = 6). (**I**) Percentage of open arm entries of the EPM (*n* = 6). (**J**) Emotionality score, a composite index derived from anxiety-related parameters in both OFT and EPM across treatment groups (*n* = 6). (**K**) Immobility time in the forced swim test (FST) (*n* = 6). (**L**) Sucrose preference ratio in the sucrose preference test (SPT) (*n* = 6). * *p* < 0.05, ** *p* < 0.01, *** *p* < 0.001, **** *p* < 0.0001 vs. control group; ## *p* < 0.01, #### *p* < 0.0001 vs. acute group. Data are expressed as mean ± SD.

Using the experimental timeline illustrated in Fig. 1A, we developed a model of METH-induced anxiety in rats. Acute METH administration significantly increased the total distance traveled in the open field test (OFT), indicative of hyperlocomotion. In contrast, chronic METH treatment further enhanced locomotor activity, suggesting the development of behavioral sensitization (Fig. 1B, C; F(2,15) = 24.15, *p* < 0.0001). Analysis of anxiety-related parameters in the OFT revealed that acute METH exposure reduced baseline anxiety, while chronic METH administration induced pronounced anxiety-like behaviors (Fig. 1D, E; D: F(2,15) = 159.6, *p* < 0.0001; E: F(2,15) = 22.11, *p* < 0.0001). Similar trends were observed in the elevated plus maze (EPM): acute METH increased the distance moved and time spent in open arms, whereas chronic METH significantly decreased these measures, reflecting heightened anxiety (Fig. 1F–I; G: F (2, 15) = 364.5, *p* < 0.0001; H: F (2, 15) = 84.20, *p* < 0.0001; I: F (2, 15) = 43.23, *p* < 0.0001). To holistically evaluate emotional states, we computed Z-emotionality scores by integrating behavioral parameters from both OFT and EPM. This analysis confirmed that acute METH exerted an anxiolytic effect, while chronic METH exacerbated anxiety-like phenotypes (Fig. 1J; F (2, 15) = 762.0, *p* < 0.0001). Neither acute nor chronic METH treatment significantly influenced depressive-like behaviors, as assessed by the sucrose preference and forced swim tests (Fig. 1K, L; K: F(2, 15) = 1.842, *p* = 0.1926; L: F(2, 15) = 0.1202, *p* = 0.8876).

**Fig. 2:**
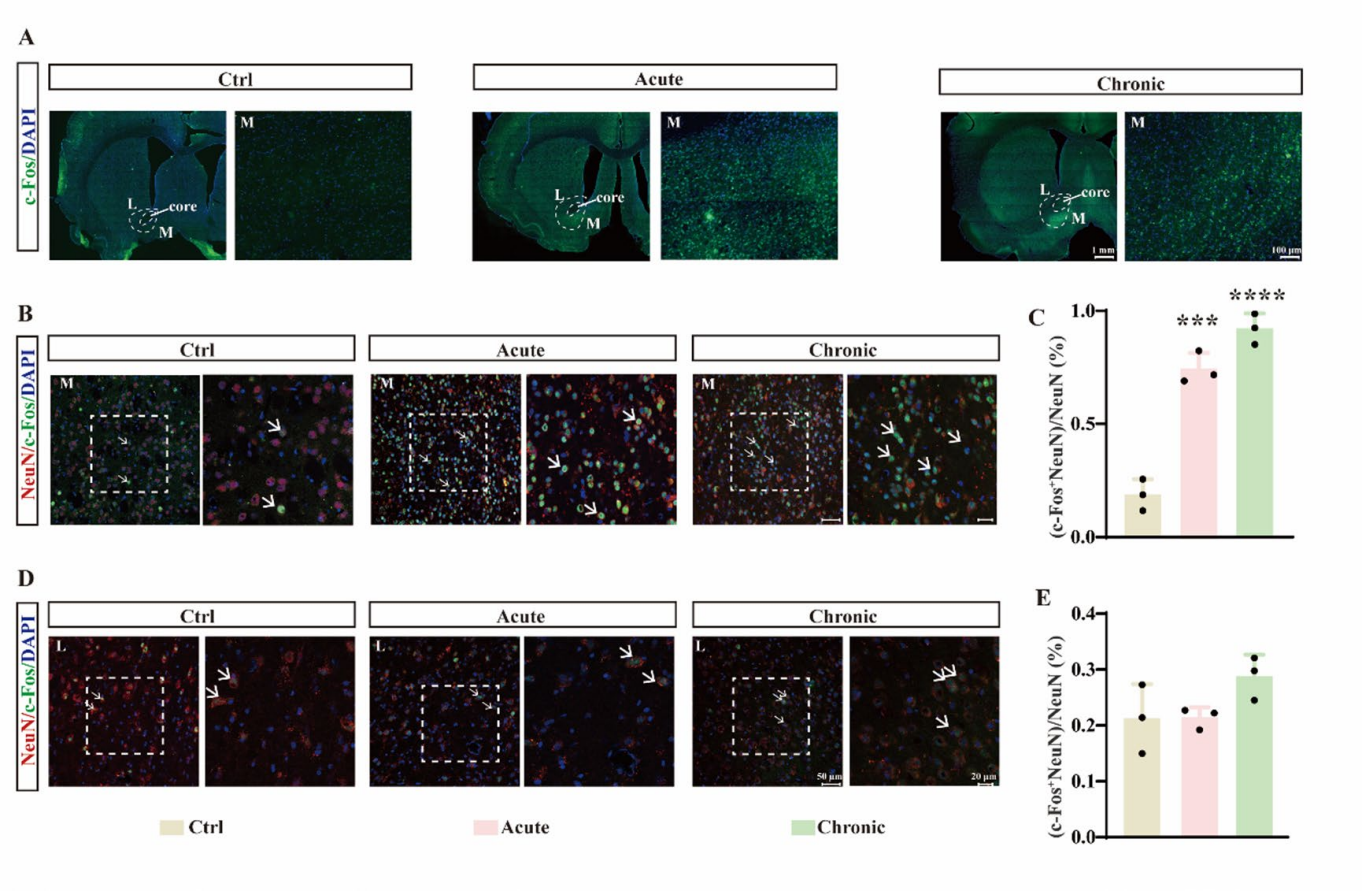
Acute and chronic METH administration significantly enhances c-Fos expression in neurons of the medial, but not lateral, shell of the rat NAc. (**A**) Representative images of c-Fos expression across the brain following acute or chronic METH treatment. L, lateral shell; M, medial shell. (**B**) Immunofluorescence staining for DAPI, NeuN, and c-Fos in the NAc medial shell. M, medial shell. (**C**) Percentage of NeuN⁺ cells co-expressing c-Fos in the medial shell. (**D**) Immunofluorescence staining for DAPI, NeuN, and c-Fos in the NAc lateral shell. L, lateral shell. (**E**) Percentage of NeuN⁺ cells co-expressing c-Fos in the lateral shell. ***p < 0.001, ****p < 0.0001 vs. control group. Data are presented as mean ± SD.

To identify neural substrates underlying these behaviors, we mapped c-Fos expression across the brain. Both acute and chronic METH robustly increased c-Fos immunoreactivity in the NAc, with more pronounced activation in the medial shell than in the lateral shell (Fig. 2A)—a regional preference consistent with the distribution of CART-positive neurons^27^. Double-labeling immunofluorescence for c-Fos and NeuN confirmed that neuronal activation was significantly elevated in the medial NAc shell following both acute and chronic METH exposure (Fig. 2B, C; F(2, 6) = 92.77, *p* < 0.0001), but not in the lateral shell (Fig. 2D, E; F (2, 6) = 2.989, *p* = 0.1257). These findings suggest that CART-positive neurons in the NAc medial shell are implicated in METH-induced behavioral sensitization and anxiety.

### METH downregulates membrane GABA_B_R expression in CART-positive neurons

**Fig. 3:**
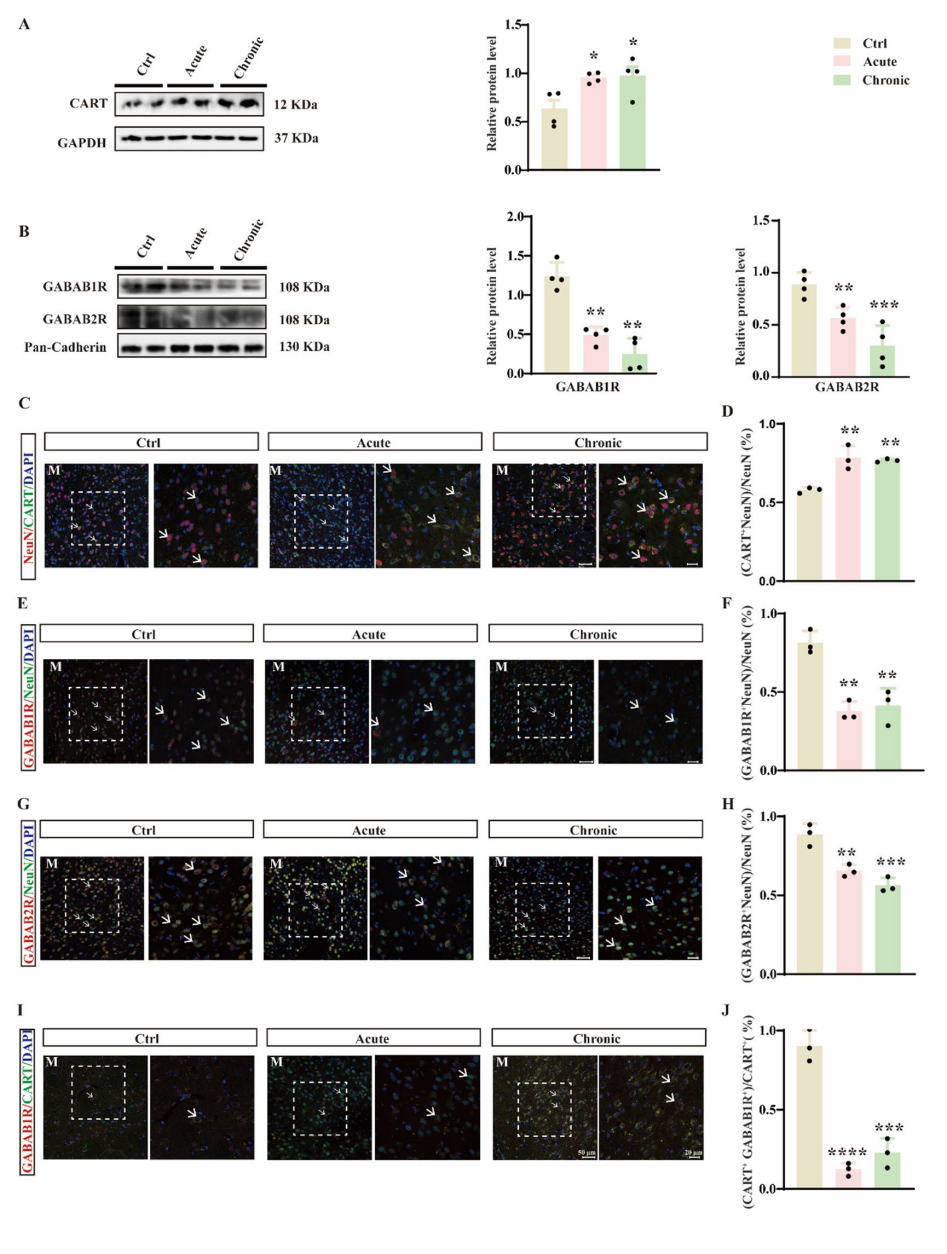
Acute and chronic METH treatments significantly elevate CART peptide expression and reduce GABA_B_R expression in CART-positive neurons. (**A**) Western blot and quantitative analysis of CART peptide in the NAc medial shell after acute and chronic METH treatments (*n* = 4). (**B**) Western blot and quantification of GABA_B1_R and GABA_B2_R expression in the NAc medial shell (*n* = 4). (**C**) Immunofluorescence staining for DAPI, NeuN, and CART in the medial shell. M, medial shell. (**D**) Proportion of NeuN⁺ cells co-positive for CART in the medial shell (*n* = 3). (**E**) Immunofluorescence staining for DAPI, NeuN, and GABA_B1_R in the medial shell. M, medial shell. (**F**) Proportion of NeuN^+^ cells co-positive for GABA_B1_R (*n* = 3). (**G**) Immunofluorescence staining for DAPI, NeuN, and GABA_B2_R in the medial shell. M, medial shell. (**H**) Proportion of NeuN^+^ cells co-positive for GABA_B2_R (*n* = 3). (**I**) Immunofluorescence staining for DAPI, CART, and GABA_B1_R in the medial shell. M, medial shell. (**J**) Proportion of CART^+^ cells co-positive for GABA_B1_R (*n* = 3). *p < 0.05, **p < 0.01, ***p < 0.001, ****p < 0.0001 vs. control group. Data are presented as mean ± SD.

We next examined the expression of CART peptide and GABA_B_R in the medial NAc shell. Western blot analysis showed that both acute and chronic METH increased CART peptide levels (Fig. 3A; F(2,9) = 6.056, *p* = 0.0216) but decreased the membrane expression of GABA_B1_R and GABA_B2_R subunits (Fig. 3B; GABA_B1_R: F(2, 9) = 37.97, *p* < 0.0001; GABA_B2_R: F(2,9) = 16.42, *p* = 0.001). Immunofluorescence staining further revealed upregulated CART expression in CART-positive neurons (Fig. 3C, D; F(2,6) = 18.11, *p* = 0.0029) and downregulated GABA_B1_R and GABA_B2_R in neurons of the medial shell (Fig. 3E–H; F: F(2,6) = 23.87, *p* = 0.0014; H: F(2,6) = 28.23, *p* = 0.0009). Colocalization studies indicated a high degree of overlap between CART and GABA_B1_R (Fig. 3I). Importantly, GABA_B1_R expression in CART-positive neurons was significantly suppressed by METH (Fig. 3J; F(2, 6) = 77.55, *p* < 0.0001).

### CART peptide injection into the NAc medial shell attenuates METH-induced behaviors

**Fig. 4:**
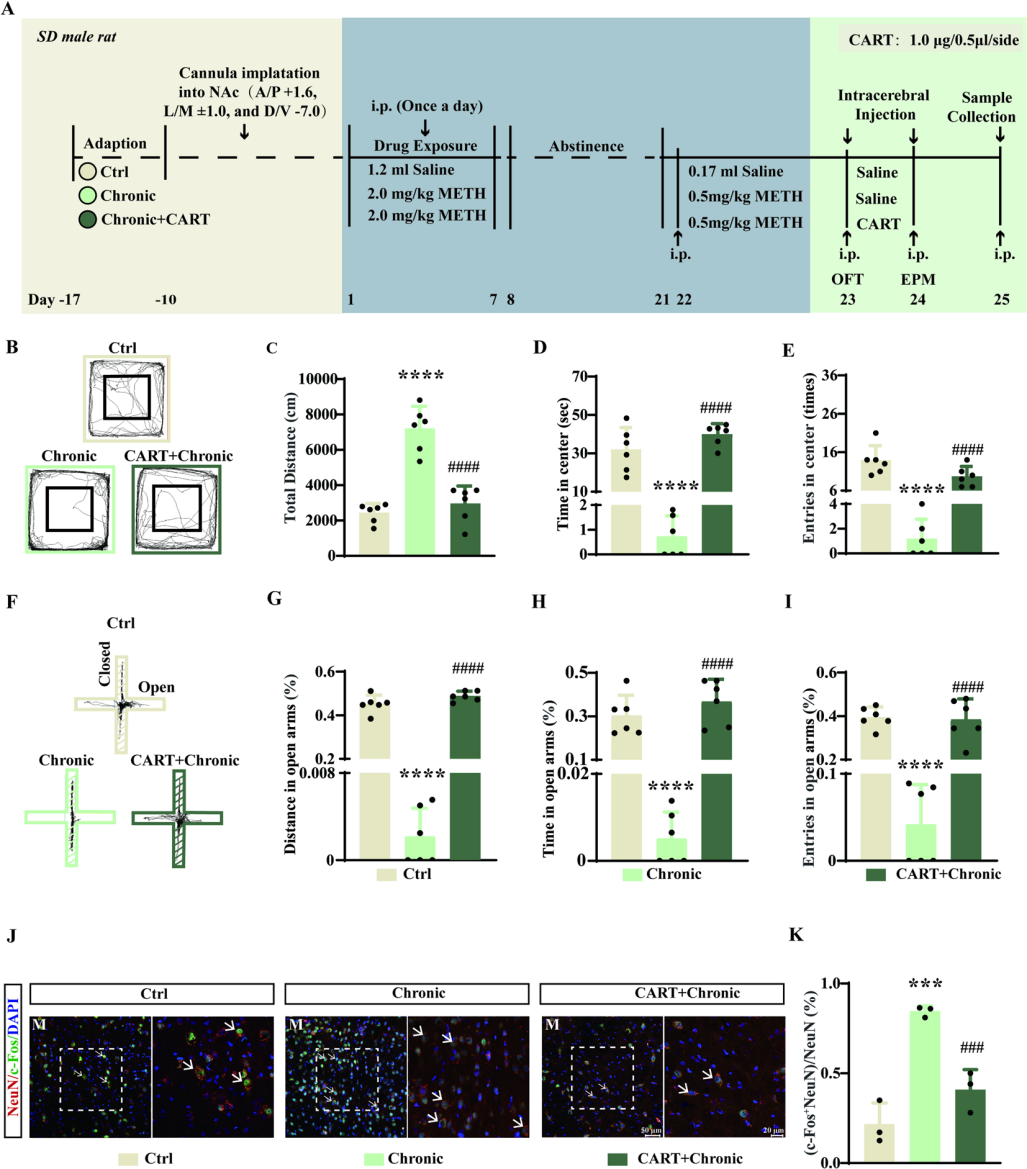
Microinjection of CART peptide into the NAc medial shell attenuates anxiety-like behaviors induced by chronic METH treatment. (**A**) Experimental timeline of chronic METH exposure and intra-NAc medial shell infusion of CART peptide. (**B**) Representative movement traces in the OFT (*n* = 6). (**C**) Total distance travelled in the OFT (*n* = 6). (**D**) Time spent in the center zone of the OFT (*n* = 6). (**E**) Number of entries into the center zone (*n* = 6). (**F**) Representative movement traces in the EPM. (**G**) Percentage of distance travelled in open arms of the EPM (*n* = 6). (**H**) Percentage of time spent in open arms (*n* = 6). (**I**) Percentage of open arm entries (*n* = 6). (**J**) Immunofluorescence staining for DAPI, NeuN, and c-Fos in the NAc medial shell. M, medial shell. (**K**) Percentage of NeuN⁺ cells co-expressing c-Fos. ***p < 0.001, ****p < 0.0001 vs. control group; ###*p* < 0.001, ####*p* < 0.0001 vs. chronic group. Data are presented as mean ± SD.

To investigate the functional role of CART, we microinjected CART peptide into the NAc medial shell of chronically METH-treated rats (Fig. 4A). CART administration significantly reduced METH-induced hyperlocomotion in the OFT (Fig. 4B, C; F(2, 15) = 42.25, *p* < 0.0001) and alleviated anxiety-like behaviors, as evidenced by increased time and entries in the center zone (Fig. 4D, E; D: F(2, 15) = 46.33, *p* < 0.0001; E: F(2,15) = 30.49, *p* < 0.0001). In the EPM, CART peptide increased open-arm entries, distance, and time spent in open arms to levels comparable to controls (Fig. 4F–I; G: F (2, 15) = 603.6, *p* < 0.0001; H: F (2, 15) = 34.83, *p* < 0.0001; I: F (2, 15) = 53.66, *p* < 0.0001). CART injection normalized METH-induced neuronal hyperactivation, as shown by reduced c-Fos expression in the medial NAc shell (Fig. 4J, K; F(2, 6) = 33.51, *p* = 0.0006).

### CART peptide restores GABA_B_R expression in CART-positive neurons

**Fig. 5:**
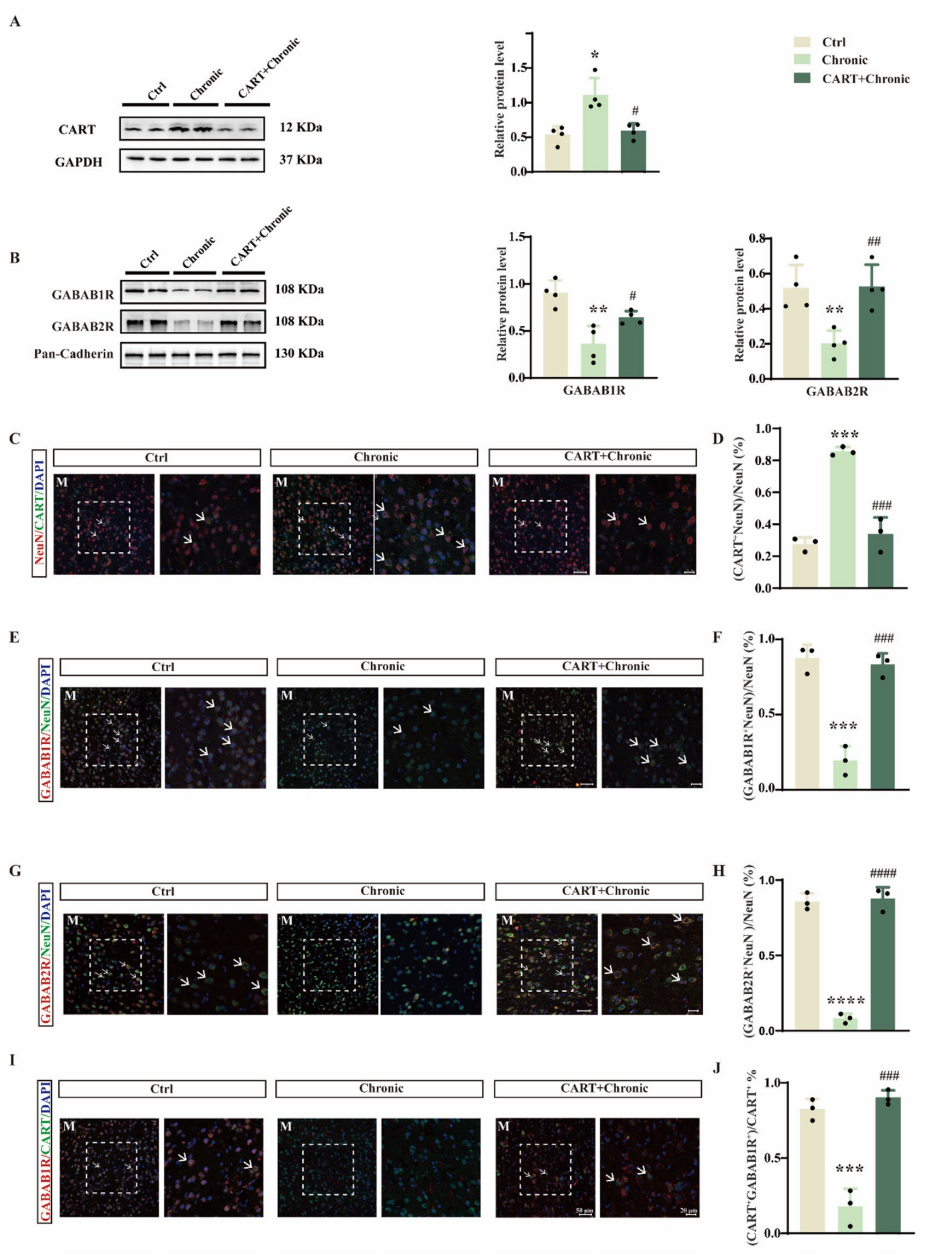
CART peptide injection into the NAc medial shell counteracts chronic METH-induced alterations in CART and GABA_B_R expression. (**A**) Western blot and quantification of CART peptide expression following chronic METH and CART peptide infusion (*n* = 4). (**B**) Western blot and quantification of GABA_B1_R and GABA_B2_R expression (*n* = 4). (**C**) Immunofluorescence staining for DAPI, NeuN, and CART in the medial shell. M, medial shell. (**D**) Percentage of NeuN^+^ cells co-expressing CART (*n* = 3). (**E**) Immunofluorescence staining for DAPI, NeuN, and GABA_B1_R. M, medial shell. (**F**) Percentage of NeuN⁺ cells co-expressing GABA_B1_R (*n* = 3). (**G**) Immunofluorescence staining for DAPI, NeuN, and GABA_B2_R. M, medial shell. (**H**) Percentage of NeuN⁺ cells co-expressing GABA_B2_R (*n* = 3). (**I**) Immunofluorescence staining for DAPI, CART, and GABA_B1_R. M, medial shell. (**J**) Percentage of CART⁺ cells co-expressing GABA_B1_R (*n* = 3). *p < 0.05, **p < 0.01, ***p < 0.001, ****p < 0.0001 vs. control group; #*p* < 0.05, ##*p* < 0.01, ###*p* < 0.001, ####*p* < 0.0001 vs. CART + Chronic group. Data are presented as mean ± SD.

We further explored the molecular mechanisms underlying CART’s behavioral effects. Western blot analysis indicated that exogenous CART peptide reversed the METH-induced upregulation of CART and the downregulation of GABA_B1_R and GABA_B2_R (Fig. 5A, B; CART: F(2, 9) = 13.60, *p* = 0.0019; GABA_B1_R: F(2, 9) = 14.48, *p* = 0.0015; GABA_B2_R: F(2, 9) = 10.53, *p* = 0.0044). Immunofluorescence confirmed these findings at the cellular level, showing decreased CART expression and restored GABA_B_R levels after CART treatment (Fig. 5C–H; D: F (2, 6) = 48.19, *p* = 0.0002; F: F (2, 6) = 56.54; *p* = 0.0001; H: F (2, 6) = 186.1, *p* < 0.0001; J: F (2, 6) = 65.59, *p* < 0.0001). Moreover, CART injection significantly increased GABA_B1_R expression in CART-positive neurons (Fig. 5I, J; F(2,6) = 65.59, *p* < 0.0001).

Molecular docking was employed to untangle the potential structural basis of the interaction between CART peptide and GABA_B_R: in the first binding segment, hydrogen bonds were formed between residues 37, 38, 39, 40, and 42 of CART peptide and residues 35, 38, 40, 41, and 42 of GABA_B1_R. In the second binding segment, hydrogen bonds were established between residues 100 and 105 of CART peptide and residues 744 and 747 of GABA_B1_R (Fig. S2A-C). Co-IP using anti-CART and anti-GABA _B1_R antibodies preliminarily suggested a potential interaction between these two proteins. (Fig. S2D).

### GABA_B_R antagonism reverses the anxiolytic effects of CART peptide

**Fig. 6:**
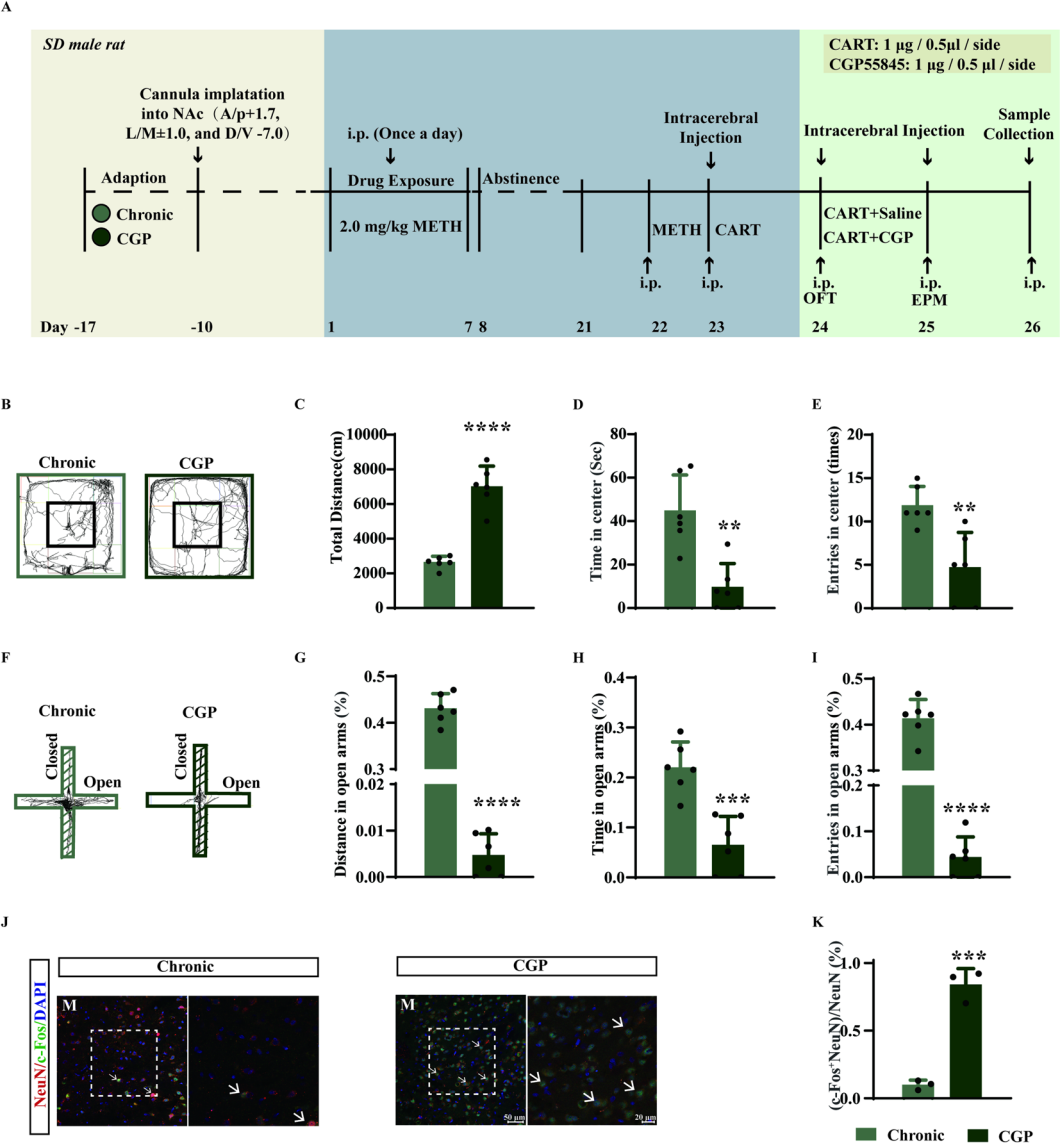
The GABA_B_R antagonist CGP55845 blocks the anxiolytic effects of CART peptide on METH-induced anxiety-like behaviors. (**A**) Experimental timeline of chronic METH treatment and intra-NAc medial shell injections of CART peptide and CGP55845. (**B**) Representative movement traces in the OFT (*n* = 6). (**C**) Total distance travelled in the OFT (*n* = 6). (**D**) Time spent in the center zone (*n* = 6). (**E**) Number of entries into the center zone (*n* = 6). (**F**) Representative movement traces in the EPM. (**G**) Percentage of distance travelled in open arms of the EPM (*n* = 6). (**H**) Percentage of time spent in open arms (*n* = 6). (**I**) Percentage of open arm entries (*n* = 6). (**J**) Immunofluorescence staining for DAPI, NeuN, and c-Fos in the medial shell. M, medial shell. (**K**) Percentage of NeuN⁺ cells co-expressing c-Fos. **p < 0.01, ***p < 0.001, ****p < 0.0001 vs. Chronic group. Data are presented as mean ± SD.

To test whether GABA_B_R mediates CART’s effects, we administered the GABA_B_R antagonist CGP55845 after CART peptide injection in METH-treated rats (Fig. 6A). CGP55845 abolished the beneficial effects of CART, reinstating behavioral sensitization (Fig. 6B, C; t (10) = 8.585, *p* < 0.0001) and anxiety-like phenotypes in the OFT (Fig. 6D, E; D: t (10) = 4.329, *p* = 0.0015; E: t (10) = 3.773, *p* = 0.0036) and EPM (Fig. 6F–I; G: t(10) = 32.21, *p* < 0.0001; H: t(10) = 4.922, *p* = 0.0006; I: t(10) = 15.01, *p* < 0.0001). Furthermore, the antagonist increased c-Fos expression in the medial NAc shell, indicating renewed neuronal activation (Fig. 6J, K; t(4) = 10.33, *p* = 0.0005). These results demonstrate that the anxiolytic and behavioral effects of CART peptide are mediated through GABA_B_R signaling.

## Discussion

**Fig. 7:**
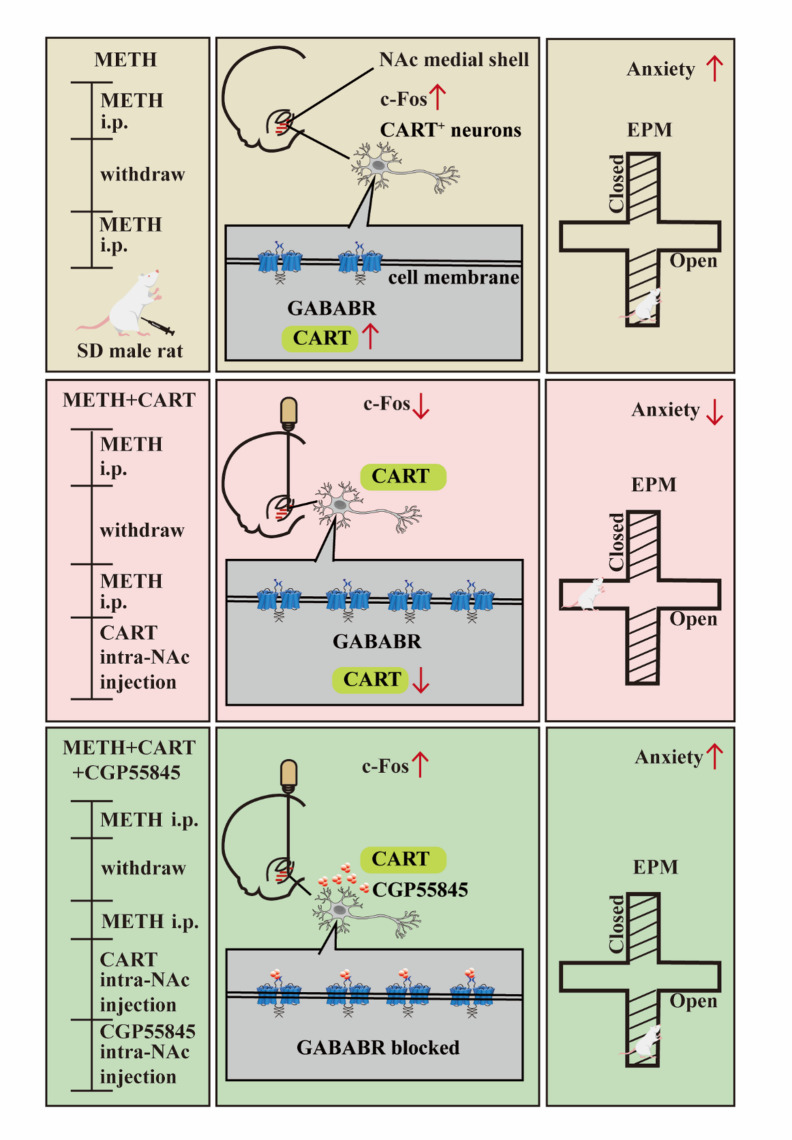
Schematic model proposing the mechanism by which intra-NAc medial shell infusion of CART peptide alleviates METH-induced anxiety-like behaviors via modulation of GABA_B_R in CART-positive neurons. Chronic METH administration activates CART-positive neurons in the NAc medial shell and downregulates GABA_B_ receptor expression, contributing to anxiety-like behaviors. CART peptide injection normalizes neuronal activation, restores GABA_B_R expression, and reduces anxiety. Antagonism of GABA_B_R with CGP55845 abolishes the protective effect of CART peptide.

The present study demonstrates that microinjection of CART peptide into the NAc medial shell attenuates METH-induced behavioral sensitization and anxiety-like behaviors by restoring GABA_B_ receptor expression in CART-positive neurons. These findings provide novel insights into the neuropeptidergic modulation of addiction-related anxiety and identify a potential mechanism through which CART peptide counteracts METH-induced psychopathology (The schematic illustration is presented in Fig. 7). While previous studies have established that intra-accumbal CART peptide reduces amphetamine-induced locomotion^18^ and cocaine self-administration^28^, its role in METH-related anxiety remained unexplored. Here, we observed that METH administration not only induced behavioral sensitization but also provoked significant anxiety-like behaviors. Immunofluorescence and western blot analyses revealed hyperactivation of NAc medial shell neurons, accompanied by elevated CART peptide expression and reduced GABA_B_R levels in CART-positive neurons, suggesting dysregulation of the endogenous CART system and aberrant GABAergic transmission. Exogenous CART peptide normalized GABA_B_R expression and alleviate METH-induced behavioral abnormalities. The combination of molecular docking and Co-IP indicates a possible interaction between CART and GABA_B_R. The therapeutic effects of exogenous CART peptide were abolished by the GABA_B_R antagonist CGP55845, further underscoring the critical role of GABA_B_R.

The NAc, particularly its shell subregion, plays a central role in reward processing, motivation, and emotional regulation^29–31^. Our data align with emerging evidence that the medial shell is especially critical in psychostimulant-induced locomotion and affective responses^32–34^. We found that METH preferentially activated the medial shell, as indicated by c-Fos expression, consistent with the regionally enriched expression of CART peptide^27^. The efficacy of intra-medial shell CART injection in reversing METH-induced behaviors further highlights the functional importance of this subregion. These findings extend prior work showing that pharmacological manipulation of dopamine receptors^35^ or neuropeptide systems^36^ within the NAc shell modulates anxiety and addiction-related behaviors. A recent study by Wang et al. revealed that METH enhances reward effects by suppressing oxytocin (OXT) signaling from the paraventricular nucleus (PVN) to the NAc core, further underscoring the functional dichotomy between the NAc core and shell in METH addiction^37^. Their results complement our observations by demonstrating that the shell subregion, where CART peptide exerts its anxiolytic effects, is functionally distinct from the core in mediating METH-related behaviors. This regional specialization should be carefully considered in developing targeted interventions.

In contrast to our findings in the NAc shell under a pathological state (METH exposure), recent work by Balasubramanian et al. (2025) reported that microinfusion of CART peptide into the dorsal raphe nucleus (DRN) of drug-naïve mice induces anxiety-like behaviors and suppresses serotonin (5-HT) neurotransmission^38^. This suggests that the functional outcomes of CART signaling are highly region-specific and state-dependent. While CART in the DRN appears to promote anxiety via inhibition of 5-HT neurons under physiological conditions, our results demonstrate that in the METH-sensitized NAc shell, CART acts on local GABAergic circuits to counteract anxiety. These divergent roles highlight the complex, context-dependent nature of CART peptide signaling and suggest that its effects are shaped by both brain region and pathological state. To further clarify the state-dependent nature of CART’s anxiolytic effects, future studies examining CART peptide injection in the NAc medial shell of drug-naïve rats will help elucidate whether its anti-anxiety action is contingent upon pathological conditions such as METH-induced GABA_B_R downregulation. Notably, the same study by Balasubramanian et al. also provided electrophysiological evidence from ex vivo patch-clamp recordings indicating that acute restraint stress selectively enhances the excitability of CART neurons in the centrally projecting Edinger–Westphal nucleus (EWcp) that project to the DRN, whereas DRN-projecting CART neurons in the NAc showed low basal excitability and were not modulated by stress^38^. This further supports the regional functional dichotomy of CART pathways: although the NAc contains CART neurons capable of projecting to the DRN, they may not be primarily engaged in stress-related responses. Instead, as our data suggest, CARTergic mechanisms within the NAc shell—particularly under psychostimulant exposure—may be more involved in restoring inhibitory control through GABAergic modulation rather than driving stress-induced excitability.

Beyond the well-characterized dopaminergic mechanisms of METH^39,40^, our study emphasizes the contribution of GABAergic signaling. Impaired GABAergic transmission is increasingly implicated in anxiety disorders^41,42^, and benzodiazepines—which enhance GABAergic activity—are commonly used to manage METH-induced anxiety^43^. Notably, NAc medial shell exhibits high expression of GABA-related genes^27^, suggesting a regional specialization for inhibitory control. In the NAc, our previous studies have demonstrated that psychostimulants trigger reduced expression of GABA_B_R on neuronal cell membranes, leading to deficits in GABAergic transmission. The underlying mechanism may involve activation of Ca^2+^/calmodulin-dependent protein kinase II (CaMKII) and its autophosphorylation at the T286 residue, which promotes phosphorylation of GABA_B_R at the S867 site, thereby inhibiting receptor recycling or facilitating lysosomal degradation and ultimately resulting in membrane receptor depletion^44^. Furthermore, the observation that knockdown of Rab10—a key molecule in vesicular transport—attenuates cocaine-induced GABA_B_R reduction suggests that psychostimulant-induced downregulation of GABA_B_R may be associated with dysregulation of the balance between receptor endocytosis and recycling^45^. We propose that CART peptide normalizes METH-induced deficits in GABAergic transmission by restoring GABA_B_R expression, thereby reducing neuronal hyperactivation and anxiety-like behaviors. This is consistent with reports that GABA_B_R activation blocks behavioral sensitization^46^ and conditioned place preference^47^, and that inhibition of GABAergic neurons rescues METH-induced anxiety^48^. The upregulation of CART after chronic METH exposure, together with our finding that exogenous CART reduces METH-induced anxiety, suggests that CART may serve as a feedback or counter-regulatory signal in the NAc medial shell. Rather than just a marker of drug exposure, this increase likely reflects an adaptive response aimed at restoring inhibitory control. In the NAc (including the medial shell), GABAergic neurons mainly comprise two types: MSNs and interneurons. Given that CART is predominantly expressed in MSNs of the NAc medial shell with minimal expression in interneurons^10,12^, and considering that MSNs constitute approximately 95% of NAc neurons while interneurons represent a relatively small proportion^27,49^, we consider that the CART effects observed in our study are more likely to be mediated through actions on MSNs. However, based on our current findings, we cannot completely exclude potential effects of CART on GABAergic interneurons, which may modulate MSNs activity through local inhibitory networks. Future studies employing cell type-specific manipulations targeting distinct cell subtypes in the NAc medial shell will help further clarify whether GABAergic interneurons contribute to CART’s actions. Furthermore, our study further elucidated from a behavioral perspective that CART alleviates METH-induced anxiety through modulation of GABA_B_R by employing the GABA_B_R-specific antagonist CGP55845. This also suggests the possibility that CGP55845 competes with CART for binding. Testing CART-GABA_B_R Co-IP in the presence or absence of CGP55845 would help determine this possibility and provide further structural insights into the potential interaction between CART and GABA_B_R.

Several limitations should be considered. While molecular docking and Co-IP preliminarily suggest a potential interaction between CART and GABA_B_R, whether CART’s regulatory effect on GABA_B_R is achieved through direct molecular interaction requires further experimental validation. Future studies employing pull-down assays with recombinant proteins and Co-IP using antibodies targeting distinct epitopes would help clarify a direct interaction between CART and GABA_B_R. Our previous work has demonstrated that both the Ca^2+^/CaMKII signaling pathway^44,50^ and the small GTPase Rab10^45^ participate in the regulation of GABA_B_R membrane expression in NAc neurons and influence behavioral sensitization induced by addictive drugs. Building on these findings, future studies should explore whether CART peptide modulates GABA_B_R via these established pathways or through other novel molecular mechanisms. Additionally, while CART-positive neurons are predominantly located in the medial shell, we cannot exclude the possibility that lateral shell CART neurons also contribute to METH-related behaviors. Future studies using optogenetic or chemogenetic approaches to selectively manipulate CART neuron activity will help elucidate their circuit-level functions. Given the current lack of pharmacokinetic studies on microinjected CART peptide in the brain, it remains undetermined whether the observed behavioral effects reflect residual pharmacological effects, longer-term neuroadaptations, or a combination of both. It is also noteworthy that CART peptide may exert anxiogenic effects in drug-naïve animals^51^, suggesting that its role is state-dependent and modulated by baseline GABA_B_R expression.

## Conclusions

In conclusion, our results indicate that microinjecting CART peptide into the medial NAc shell mitigates METH-induced behavioral sensitization and anxiety by restoring GABA_B_R expression and function. These findings not only advance our understanding of peptidergic regulation in addiction and anxiety but also support the therapeutic potential of targeting the CART–GABA_B_R system in METH use disorder.

## Materials and methods

### Animals

Male Sprague-Dawley rats (approximately 250 g) purchased from Hunan Silaikejingda Experimental Animal Co., Ltd. (Changsha, China) were used in this study. After a one-week acclimatization period, rats were maintained under a 12 h/12 h light/dark cycle with ad libitum access to food and water. All experimental procedures were approved by the Animal Experimentation Ethics Committee of Nanchang University (Permit Number: NCULAE-20220624033) and conducted in accordance with the National Institutes of Health Guide for the Care and Use of Laboratory Animals. This study was reported in accordance with ARRIVE guidelines. All behavioral tests were conducted at the same time each day (09:00–13:00; Zeitgeber Time 2–6) to minimize circadian influences.

### Drugs and reagents

Methamphetamine (METH), baclofen, CGP55845 and pentobarbital sodium was purchased from Sigma-Aldrich, and CART peptide was obtained from Phoenix Pharmaceuticals. CART peptide was dissolved in saline to prepare a 2 µg/µl CART solution. CGP55845 was first dissolved in dimethyl sulfoxide (DMSO) and subsequently diluted in saline to achieve a final concentration of 2 µg/µl. Saline containing an equivalent concentration of DMSO was used for the saline group. For Western blot and Co-IP, the following antibodies were used: anti-CART (YN2156, ImmunoWay), anti–Pan-Cadherin (YM8426, ImmunoWay), anti-GAPDH (60004-1-Ig, Proteintech), mouse IgG (B900620, Proteintech), anti-GABA_B1_R (sc-398901, Santa Cruz), and anti-GABA_B2_R (sc-393270, Santa Cruz). For immunofluorescence, primary antibodies included anti–c-Fos (226008, Synaptic Systems), anti-NeuN (AB104224, Abcam), anti-CART (DF15431, Affinity), anti-GABA_B1_R (AB5505, Abcam), and anti-GABA_B2_R (AB75838, Abcam). Fluorescent and horseradish peroxidase (HRP)-conjugated secondary antibodies were from Proteintech.

### Experimental design

Rats were randomly assigned to experimental groups using a computer-generated randomization sequence. The study consisted of three phases:

#### METH withdrawal and reinstatement

Rats received intraperitoneal (i.p.) injections of either METH (2 mg/kg in 2 mL/kg saline) or saline for 7 consecutive days (once per day). After a 14-day withdrawal period, rats were challenged with METH (0.5 mg/kg) or saline 20 min before all behavioral tests. Groups included: Chronic (METH during exposure and challenge), Acute (saline during exposure, METH challenge), and Control (saline throughout). All rats except those in the control group were administered an intraperitoneal injection of 0.5 mg/kg METH 20 min prior to sample collection.

#### Anxiety-like behavior and CART intervention

Rats were divided into Control, Chronic, and CART + Chronic groups. Both Control and Chronic groups received bilateral microinjections of saline (0.5 µL per side) into the NAc medial shell following the withdrawal and reinstatement. The CART + Chronic group received bilateral infusions of CART peptide (1 µg/0.5 µL per side) into the same region. Two hours later, the rats received an intraperitoneal injection of METH and underwent behavioral testing 20 min thereafter. All rats except those in the control group were administered an intraperitoneal injection of 0.5 mg/kg METH 20 min prior to sample collection.

#### GABA_B_R antagonism

To determine whether CART’s effects were mediated via GABA_B_R, an additional antagonism experiment was performed. Following METH withdrawal and reinstatement, rats first received CART microinjection into the NAc medial shell as described above to establish the same chronic METH treatment-CART injection model. Over the next two days, rats in the CGP group received a microinjection of the GABA_B_R antagonist CGP55845 (1 µg/0.5 µL per side) 2 h after CART peptide injection. Chronic animals received saline (0.5 µL per side) instead of CGP55845 at the corresponding time points. Two hours after the administration of CGP55845 or saline, the animals received an intraperitoneal injection of 0.5 mg/kg METH, and behavioral testing was conducted 20 min later. On the day following the completion of behavioral testing, rats were administered an intraperitoneal injection of 0.5 mg/kg METH 20 min prior to sample collection.

### Surgery and intra-NAc injection

Rats were anesthetized with pentobarbital sodium (50 mg/kg, i.p.) and placed in a stereotaxic apparatus. Guide cannulas (22 gauge; Plastics One) were implanted bilaterally into the NAc shell (coordinates: AP + 1.6 mm, ML ± 1.0 mm, DV − 7.0 mm from bregma). The cannulas were secured with dental cement and fitted with dust caps. Following surgery, rats were housed individually to prevent cannula displacement, wound infection, and potential brain damage caused by licking or fighting among cage mates. Cannula placement was verified by Nissl staining (Fig. S1). Rats were given 10 days to recover from surgery. For intra-NAc injection, CART peptide (1 µg/0.5 µL per side) and/or CGP55845 (1 µg/0.5 µL per side) were infused via an injection needle (28 gauge; Plastics One) connected to a 5 µL syringe (Neurostar Co.) and manually perfused at a rate of 0.2 µL/min. After the injection was completed, the injector needle was left in place for five minutes to allow for solution diffusion.

### Behavioral tests

All behavioral tests were recorded and analyzed using SMART 3.0 software.

#### Open field test

Rats were placed in a 100 × 100 × 50 cm arena for 10 min after 30 min habituation. Total distance traveled and time in center were measured. The arena was cleaned with 75% ethanol between trials.

#### Elevated plus maze

Rats were placed in the center of a plus-shaped maze with two open and two closed arms (50 × 10 cm) 60 cm above the floor and allowed to explore for 5 min. Time spent in open arms was recorded.

#### Forced swim test

Rats were placed in a cylindrical tank (40 cm diameter, 75 cm height) filled with water (25 °C) to a depth of 25 cm for 6 min. Immobility time during the last 4 min was analyzed.

#### Sucrose preference test

After 24 h acclimation to two bottles (1% sucrose and water), rats were deprived of food and water for 12 h, then presented with pre-weighed bottles for 12 h. Sucrose preference was calculated as (sucrose consumption / total fluid intake) × 100%.

### Immunofluorescence

For the immunofluorescence assay, an independent cohort of animals that underwent the identical drug treatment paradigm was employed. Rats were euthanized by an overdose of pentobarbital sodium (0.8 g/kg, i.p.) and transcardially perfused with 0.9% sodium chloride followed by 4% paraformaldehyde (PFA). Brains were carefully removed and post-fixed in 4% PFA for 24 h, then cryoprotected in 30% sucrose solution. Subsequently, the brains were embedded in optimal cutting temperature (OCT) compound (Sakura Finetek, Torrance, CA, USA) and sectioned coronally at 20–50 μm thickness using a freezing microtome (Leica CM 1950, Leica Biosystems, Wetzlar, Germany). The 50-µm sections were used for whole-brain c-Fos mapping, while 20-µm sections were employed for standard immunofluorescence staining.

Sections were processed as free-floating sections and were permeabilized with 0.1% Triton X-100 in PBS and blocked with goat serum. They were then incubated overnight at 4 °C with the following primary antibodies: anti-NeuN (1:150), anti-c-Fos (1:1000), anti-CART (1:150), anti-GABA_B1_R (1:150), and anti-GABA_B2_R (1:150). After washing, sections were incubated with corresponding fluorescent secondary antibodies (1:400) at 37 °C for 1 h. After additional PBS washes, sections were transferred onto gelatin-coated slides and mounted using an anti-fade mounting medium containing DAPI. Images were acquired using a confocal microscope (ZEISS LSM980, Oberkochen, Germany) for high-resolution fluorescence visualization. Whole-brain c-Fos expression was captured with a digital slide scanner (Olympus VS200, Tokyo, Japan). Neuronal nuclei were identified via immunostaining for neuronal nuclear protein (NeuN). To quantify neuronal expression, the percentage of NeuN-positive cells that were also positive for c-Fos, GABA_B1_R, GABA_B2_R, or CART was calculated. Similarly, the proportion of CART-positive cells expressing GABA_B1_R was determined by counting double-labeled cells among all CART-immunoreactive neurons.

### Western Blotting

Tissue samples from the NAc were homogenized in RIPA lysis buffer supplemented with protease and phosphatase inhibitors (CWBiotech). Cytosolic and membrane protein fractions were isolated using a membrane protein extraction kit (k268-50, BioVision) according to the manufacturer’s instructions. Protein concentration was determined using a BCA assay kit (Beyotime Biotechnology). Equal amounts of protein were separated by 10% SDS-PAGE and transferred to polyvinylidene fluoride (PVDF) membranes. After blocking with 5% skimmed milk for 2 h at room temperature, the membranes were incubated overnight at 4 °C with the following primary antibodies: anti-GABA_B1_R (1:1000), anti-GABA_B2_R (1:1000), anti-CART (1:1000), with anti-GAPDH (1:2000) and anti–Pan-Cadherin (1:2000) serving as loading controls for cytosolic and membrane fractions, respectively. Subsequently, membranes were incubated with HRP-conjugated secondary antibodies (1:10,000) for 1.5 h at room temperature. Protein bands were visualized using an eECL Western Blot Kit (CWBiotech) and quantified with ImageJ software. The relative expression level of the target protein was calculated as the ratio of its band intensity to that of the internal reference protein.

### Molecular docking analysis

The amino acid sequences of GABA_B1_R (ID: Q9Z0U4), GABA_B2_R (ID: O88871), and CART (ID: P49192) were obtained from the UniProt database. The structures and interactions of GABA_B_R and CART were predicted based on their sequences using AlphaFold3^52^, and the interaction sites were analyzed with PyMOL v2.5.2.

### Co-immunoprecipitation

Co-IP was conducted to detect protein interactions. Specifically, we took 2 mg of lysate from the total protein of the rat NAc medial shell, added 3 µg of specific antibody (anti-GABAB1R (sc-398901, Santa Cruz) or anti-CART (YN2156, ImmunoWay), and rotated it overnight at 4 °C. An equal amount of protein lysate and IgG were used as a negative control group, which was incubated under the same conditions. The samples obtained from the above steps were mixed with Protein G beads and rotated at 4 °C for 3 h, then washed five times with PBS. Samples were then incubated in loading buffer at 100 °C for 5 min. Subsequently, Western blot experiments were performed to detect different proteins in the samples. Both negative (IgG) and positive (Input) control groups were established.

### Statistical analysis

Data were analyzed using GraphPad Prism 6. One-way ANOVA with Tukey’s or Dunnett’s post hoc test and unpaired t-tests were used where appropriate. Emotionality Z-scores were calculated as described by Guilloux et al.^53^. Data are presented as mean ± SD; *p* < 0.05 was considered significant.

## Supplementary Information

Below is the link to the electronic supplementary material.

Supplementary Material 1

## Data Availability

The datasets generated and/or analyzed during this study are available from the corresponding author upon reasonable request.

## References

[1] Farrell, M. et al. Responding to global stimulant use: challenges and opportunities. *Lancet (London England)*. **394**, 1652–1667. 10.1016/s0140-6736(19)32230-5 (2019). 31668409 10.1016/S0140-6736(19)32230-5 PMC6924572

[2] Son, Y. et al. Global prevalence of cannabis and amphetamine/methamphetamine use among adolescents in 47 countries: a population-based study from WHO database. *World J. pediatrics: WJP*. **21**, 291–305. 10.1007/s12519-025-00883-w (2025). 40108046 10.1007/s12519-025-00883-w

[3] Darke, S., Kaye, S., McKetin, R. & Duflou, J. Major physical and psychological harms of methamphetamine use. *Drug Alcohol Rev.* **27**, 253–262. 10.1080/09595230801923702 (2008). 18368606 10.1080/09595230801923702

[4] Shi, S., Sun, Y., Zan, G. & Zhao, M. The interaction between central and peripheral immune systems in methamphetamine use disorder: current status and future directions. *J. Neuroinflamm.* **22**, 40. 10.1186/s12974-025-03372-z (2025). 10.1186/s12974-025-03372-z PMC11829452 39955589

[5] Li, J. H. et al. Gut microbiota mediates prenatal METH exposure-induced anxiety- and depression-like behaviors by modulating the Wnt signaling pathway. *Brain. Behav. Immun.* **130**, 106112. 10.1016/j.bbi.2025.106112 (2025). 40976406 10.1016/j.bbi.2025.106112

[6] Mohtashami, T., Atashi, A., Garmabi, B. & Khaksari, M. Neuroprotective Effects of Platelet-Derived Exosomes in a Rat Model of Methamphetamine-Induced Neurotoxicity. *Mol. Neurobiol.* **63**, 59. 10.1007/s12035-025-05382-7 (2025). 41247551 10.1007/s12035-025-05382-7

[7] Wang, X. et al. Indole derivatives ameliorated the methamphetamine-induced depression and anxiety via aryl hydrocarbon receptor along microbiota-brain axis. *Gut microbes*. **17**, 2470386. 10.1080/19490976.2025.2470386 (2025). 39996473 10.1080/19490976.2025.2470386 PMC11864316

[8] Yates, J. R. Pharmacological Treatments for Methamphetamine Use Disorder: Current Status and Future Targets. *Subst. abuse rehabilitation*. **15**, 125–161. 10.2147/sar.S431273 (2024). 10.2147/SAR.S431273 PMC11370775 39228432

[9] Rogge, G., Jones, D., Hubert, G. W., Lin, Y. & Kuhar, M. J. CART peptides: regulators of body weight, reward and other functions. *Nat. Rev. Neurosci.* **9**, 747–758. 10.1038/nrn2493 (2008). 18802445 10.1038/nrn2493 PMC4418456

[10] Smith, Y., Kieval, J., Couceyro, P. R. & Kuhar, M. J. CART peptide-immunoreactive neurones in the nucleus accumbens in monkeys: ultrastructural analysis, colocalization studies, and synaptic interactions with dopaminergic afferents. *J. Comp. Neurol.* **407**, 491–511. 10.1002/(sici)1096-9861(19990517)407:4<491::aid-cne3>3.0.co;2-0 (1999). 10235641 10.1002/(sici)1096-9861(19990517)407:4<491::aid-cne3>3.0.co;2-0

[11] Yang, S. C., Shieh, K. R. & Li, H. Y. Cocaine- and amphetamine-regulated transcript in the nucleus accumbens participates in the regulation of feeding behavior in rats. *Neuroscience* **133**, 841–851. 10.1016/j.neuroscience.2005.03.023 (2005). 15908130 10.1016/j.neuroscience.2005.03.023

[12] Kuhar, M. J., Jaworski, J. N., Hubert, G. W., Philpot, K. B. & Dominguez, G. Cocaine- and amphetamine-regulated transcript peptides play a role in drug abuse and are potential therapeutic targets. *AAPS J.* **7**, E259–265. 10.1208/aapsj070125 (2005). 16146347 10.1208/aapsj070125 PMC2751515

[13] Hubert, G. W. & Kuhar, M. J. Cocaine administration increases the fraction of CART cells in the rat nucleus accumbens that co-immunostain for c-Fos. *Neuropeptides* **42**, 339–343. 10.1016/j.npep.2008.01.001 (2008). 18314190 10.1016/j.npep.2008.01.001 PMC2493299

[14] del Miraglia, E. et al. Adolescents carrying a missense mutation in the CART gene exhibit increased anxiety and depression. *Depress. Anxiety*. **23**, 90–92. 10.1002/da.20156 (2006). 16400624 10.1002/da.20156

[15] Asakawa, A. et al. Cocaine-amphetamine-regulated transcript influences energy metabolism, anxiety and gastric emptying in mice. *Hormone metabolic Res. = Hormon- und Stoffwechselforschung = Horm. et Metab.* **33**, 554–558. 10.1055/s-2001-17205 (2001). 10.1055/s-2001-17205 11561216

[16] Chaki, S., Kawashima, N., Suzuki, Y., Shimazaki, T. & Okuyama, S. Cocaine- and amphetamine-regulated transcript peptide produces anxiety-like behavior in rodents. *Eur. J. Pharmacol.* **464**, 49–54. 10.1016/s0014-2999(03)01368-2 (2003). 12600694 10.1016/s0014-2999(03)01368-2

[17] Jaworski, J. N., Kozel, M. A., Philpot, K. B. & Kuhar, M. J. Intra-accumbal injection of CART (cocaine-amphetamine regulated transcript) peptide reduces cocaine-induced locomotor activity. *J. Pharmacol. Exp. Ther.* **307**, 1038–1044. 10.1124/jpet.103.052332 (2003). 14551286 10.1124/jpet.103.052332

[18] Kim, J. H., Creekmore, E. & Vezina, P. Microinjection of CART peptide 55–102 into the nucleus accumbens blocks amphetamine-induced locomotion. *Neuropeptides* **37**, 369–373. 10.1016/j.npep.2003.10.001 (2003). 14698680 10.1016/j.npep.2003.10.001

[19] Jones, K. A. et al. GABA(B) receptors function as a heteromeric assembly of the subunits GABA(B)R1 and GABA(B)R2. *Nature* **396**, 674–679. 10.1038/25348 (1998). 9872315 10.1038/25348

[20] Pagano, A. et al. C-terminal interaction is essential for surface trafficking but not for heteromeric assembly of GABA(b) receptors. *J. neuroscience: official J. Soc. Neurosci.* **21**, 1189–1202. 10.1523/jneurosci.21-04-01189.2001 (2001). 10.1523/JNEUROSCI.21-04-01189.2001 PMC6762227 11160389

[21] Xu, C., Zhang, W., Rondard, P., Pin, J. P. & Liu, J. Complex GABAB receptor complexes: how to generate multiple functionally distinct units from a single receptor. *Front. Pharmacol.* **5**10.3389/fphar.2014.00012 (2014). 10.3389/fphar.2014.00012 PMC3920572 24575041

[22] Cryan, J. F. & Kaupmann, K. Don’t worry ‘B’ happy! a role for GABA(B) receptors in anxiety and depression. *Trends Pharmacol. Sci.* **26**, 36–43. 10.1016/j.tips.2004.11.004 (2005). 15629203 10.1016/j.tips.2004.11.004

[23] Phillips, T. J. & Reed, C. Targeting GABAB receptors for anti-abuse drug discovery. *Expert Opin. Drug Discov.* **9**, 1307–1317. 10.1517/17460441.2014.956076 (2014). 25195620 10.1517/17460441.2014.956076

[24] Ni, T. et al. Medial prefrontal cortex Notch1 signalling mediates methamphetamine-induced psychosis via Hes1-dependent suppression of GABA(B1) receptor expression. *Mol. Psychiatry*. **27**, 4009–4022. 10.1038/s41380-022-01662-z (2022). 35732696 10.1038/s41380-022-01662-z PMC9718672

[25] Lopes, A. P. et al. GABAA and GABAB agonist microinjections into medial accumbens shell increase feeding and induce anxiolysis in an animal model of anxiety. *Behav. Brain. Res.* **184**, 142–149. 10.1016/j.bbr.2007.07.001 (2007). 17714798 10.1016/j.bbr.2007.07.001

[26] Cryan, J. F. et al. Behavioral characterization of the novel GABAB receptor-positive modulator GS39783 (N,N’-dicyclopentyl-2-methylsulfanyl-5-nitro-pyrimidine-4,6-diamine): anxiolytic-like activity without side effects associated with baclofen or benzodiazepines. *J. Pharmacol. Exp. Ther.* **310**, 952–963. 10.1124/jpet.104.066753 (2004). 15113848 10.1124/jpet.104.066753

[27] Chen, G. et al. Distinct reward processing by subregions of the nucleus accumbens. *Cell. Rep.* **42**, 112069. 10.1016/j.celrep.2023.112069 (2023). 36753418 10.1016/j.celrep.2023.112069

[28] Jaworski, J. N., Hansen, S. T., Kuhar, M. J. & Mark, G. P. Injection of CART (cocaine- and amphetamine-regulated transcript) peptide into the nucleus accumbens reduces cocaine self-administration in rats. *Behav. Brain. Res.* **191**, 266–271. 10.1016/j.bbr.2008.03.039 (2008). 18485497 10.1016/j.bbr.2008.03.039 PMC2497435

[29] Bewernick, B. H. et al. Nucleus accumbens deep brain stimulation decreases ratings of depression and anxiety in treatment-resistant depression. *Biol. Psychiatry*. **67**, 110–116. 10.1016/j.biopsych.2009.09.013 (2010). 19914605 10.1016/j.biopsych.2009.09.013

[30] Salgado, S. & Kaplitt, M. G. The Nucleus Accumbens: A Comprehensive Review. *Stereotact. Funct. Neurosurg.* **93**, 75–93. 10.1159/000368279 (2015). 25720819 10.1159/000368279

[31] Volkow, N. D., Michaelides, M. & Baler, R. The Neuroscience of Drug Reward and Addiction. *Physiol. Rev.* **99**, 2115–2140. 10.1152/physrev.00014.2018 (2019). 31507244 10.1152/physrev.00014.2018 PMC6890985

[32] Ikemoto, S. Ventral striatal anatomy of locomotor activity induced by cocaine, D-amphetamine, dopamine and D1/D2 agonists. *Neuroscience* **113**, 939–955. 10.1016/s0306-4522(02)00247-6 (2002). 12182899 10.1016/s0306-4522(02)00247-6

[33] Ikemoto, S. Dopamine reward circuitry: two projection systems from the ventral midbrain to the nucleus accumbens-olfactory tubercle complex. *Brain Res. Rev.* **56**, 27–78. 10.1016/j.brainresrev.2007.05.004 (2007). 17574681 10.1016/j.brainresrev.2007.05.004 PMC2134972

[34] Vachez, Y. M. et al. Ventral arkypallidal neurons inhibit accumbal firing to promote reward consumption. *Nat. Neurosci.* **24**, 379–390. 10.1038/s41593-020-00772-7 (2021). 33495635 10.1038/s41593-020-00772-7 PMC7933121

[35] Ahmadi, H., Nasehi, M., Rostami, P. & Zarrindast, M. R. Involvement of the nucleus accumbens shell dopaminergic system in prelimbic NMDA-induced anxiolytic-like behaviors. *Neuropharmacology* **71**, 112–123. 10.1016/j.neuropharm.2013.03.017 (2013). 23566820 10.1016/j.neuropharm.2013.03.017

[36] Sun, L. L. et al. Role of melanin-concentrating hormone in the nucleus accumbens shell in rats behaviourally sensitized to methamphetamine. *Int. J. Neuropsychopharmacol.* **16**, 1767–1780. 10.1017/s1461145713000072 (2013). 23449013 10.1017/S1461145713000072

[37] Cheng, Y. J. et al. Oxytocinergic input from the paraventricular nucleus to the nucleus accumbens core modulates methamphetamine-conditioned place preference. *Nat. Commun.* **16**, 4808. 10.1038/s41467-025-59859-z (2025). 40410135 10.1038/s41467-025-59859-z PMC12102147

[38] Balasubramanian, N. et al. A New Insight into the Role of CART Peptide in Serotonergic Function and Anxiety. *J. neuroscience: official J. Soc. Neurosci.* **45**10.1523/jneurosci.0467-24.2024 (2025). 10.1523/JNEUROSCI.0467-24.2024 PMC11800755 39909575

[39] Hadlock, G. C., Chu, P. W., Walters, E. T., Hanson, G. R. & Fleckenstein, A. E. Methamphetamine-induced dopamine transporter complex formation and dopaminergic deficits: the role of D2 receptor activation. *J. Pharmacol. Exp. Ther.* **335**, 207–212. 10.1124/jpet.110.166660 (2010). 20622144 10.1124/jpet.110.166660 PMC2957782

[40] Vanderschuren, L. J. & Kalivas, P. W. Alterations in dopaminergic and glutamatergic transmission in the induction and expression of behavioral sensitization: a critical review of preclinical studies. *Psychopharmacology* **151**, 99–120. 10.1007/s002130000493 (2000). 10972458 10.1007/s002130000493

[41] Babaev, O. & Piletti Chatain, C. Krueger-Burg, D. Inhibition in the amygdala anxiety circuitry. *Exp. Mol. Med.* **50**, 1–16. 10.1038/s12276-018-0063-8 (2018). 29628509 10.1038/s12276-018-0063-8 PMC5938054

[42] Nuss, P. Anxiety disorders and GABA neurotransmission: a disturbance of modulation. *Neuropsychiatr. Dis. Treat.* **11**, 165–175. 10.2147/ndt.S58841 (2015). 25653526 10.2147/NDT.S58841 PMC4303399

[43] Glasner-Edwards, S. & Mooney, L. J. Methamphetamine psychosis: epidemiology and management. *CNS drugs*. **28**, 1115–1126. 10.1007/s40263-014-0209-8 (2014). 25373627 10.1007/s40263-014-0209-8 PMC5027896

[44] Lu, M. F. et al. The CaMKII-dependent phosphorylation of GABA(B) receptors in the nucleus accumbens was involved in cocaine-induced behavioral sensitization in rats. *CNS Neurosci. Ther.* **29**, 1345–1356. 10.1111/cns.14107 (2023). 36756679 10.1111/cns.14107 PMC10068462

[45] Yu, Z. et al. Role of Rab10 in cocaine-induced behavioral effects is associated with GABAB receptor membrane expression in the nucleus accumbens. *Front. Pharmacol.* **15**, 1496657. 10.3389/fphar.2024.1496657 (2024). 39669198 10.3389/fphar.2024.1496657 PMC11635607

[46] Bartoletti, M., Gubellini, C., Ricci, F. & Gaiardi, M. Baclofen blocks the development of sensitization to the locomotor stimulant effect of amphetamine. *Behav. Pharmacol.* **16**, 553–558. 10.1097/01.fbp.0000179279.98029.e9 (2005). 16170232 10.1097/01.fbp.0000179279.98029.e9

[47] Halbout, B., Quarta, D., Valerio, E., Heidbreder, C. A. & Hutcheson, D. M. The GABA-B positive modulator GS39783 decreases psychostimulant conditioned-reinforcement and conditioned-reward. *Addict. Biol.* **16**, 416–427. 10.1111/j.1369-1600.2010.00278.x (2011). 21309927 10.1111/j.1369-1600.2010.00278.x

[48] Xu, X. et al. Specific Inhibition of Interpeduncular Nucleus GABAergic Neurons Alleviates Anxiety-Like Behaviors in Male Mice after Prolonged Abstinence from Methamphetamine. *J. neuroscience: official J. Soc. Neurosci.* **43**, 803–811. 10.1523/jneurosci.1767-22.2022 (2023). 10.1523/JNEUROSCI.1767-22.2022 PMC9899084 36564185

[49] Tepper, J. M., Tecuapetla, F. & Koós, T. Ibáñez-Sandoval, O. Heterogeneity and diversity of striatal GABAergic interneurons. *Front Neuroanat.* **4**, 150. 10.3389/fnana.2010.00150 (2010). 21228905 10.3389/fnana.2010.00150 PMC3016690

[50] Xiong, L. et al. Cocaine- and amphetamine-regulated transcript peptide in the nucleus accumbens shell inhibits cocaine-induced locomotor sensitization to transient over-expression of α-Ca(2+) /calmodulin-dependent protein kinase II. *J. Neurochem.* **146**, 289–303. 10.1111/jnc.14289 (2018). 29313985 10.1111/jnc.14289

[51] Stanek, L. M. Cocaine- and amphetamine related transcript (CART) and anxiety. *Peptides* **27**, 2005–2011. 10.1016/j.peptides.2006.01.027 (2006). 16774797 10.1016/j.peptides.2006.01.027

[52] Abramson, J. et al. Accurate structure prediction of biomolecular interactions with AlphaFold 3. *Nature* **630**, 493–500. 10.1038/s41586-024-07487-w (2024). 38718835 10.1038/s41586-024-07487-w PMC11168924

[53] Guilloux, J. P., Seney, M., Edgar, N. & Sibille, E. Integrated behavioral z-scoring increases the sensitivity and reliability of behavioral phenotyping in mice: relevance to emotionality and sex. *J. Neurosci. Methods*. **197**, 21–31. 10.1016/j.jneumeth.2011.01.019 (2011). 21277897 10.1016/j.jneumeth.2011.01.019 PMC3086134

