# Supplementary material for: Microinjection of CART peptide into the nucleus accumbens medial shell attenuates methamphetamine-induced anxiety-like behaviors via restoration of GABAB receptor membrane expression
Source: Sci Rep. 2026 Mar 29;16:10719. doi: 10.1038/s41598-026-46389-x (PMC13039751; doi:10.1038/s41598-026-46389-x)

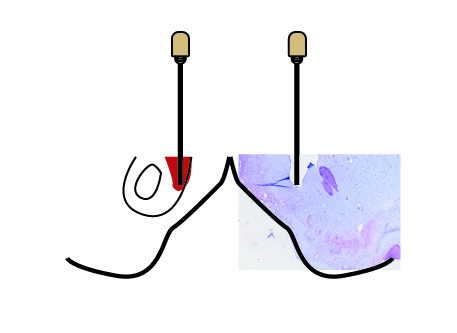


**Fig. S1** Cannula placement was verified by Nissl staining to confirm anatomical localization within the target region.


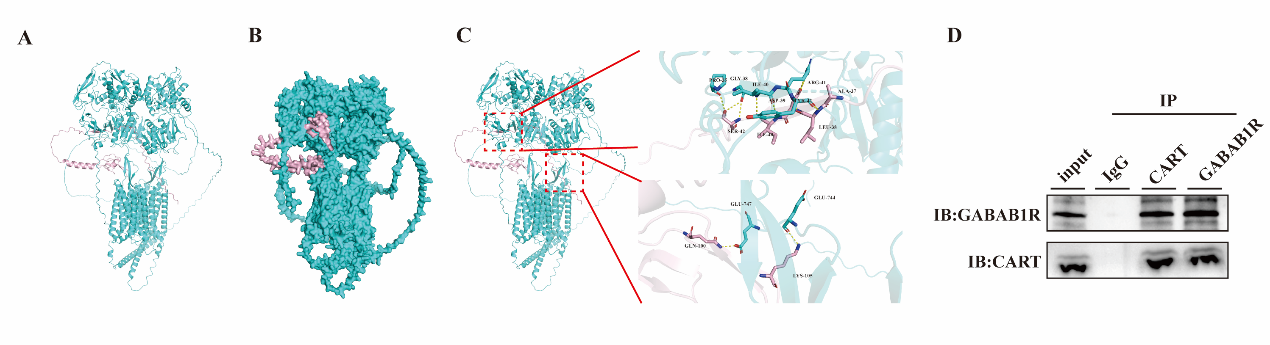


**Fig. S2.** **Assessment of a potential interaction between GABA_B_R and CART peptide** (**A**) Predicted structure of the GABA_B_R-CART peptide complex by AlphaFold3. (**B**) Interaction interface analysis of the complex using PyMOL. (**C**) Key residue pairs mediating GABA_B_R-CART peptide interaction predicted by PyMOL. (**D**) Co-immunoprecipitation (Co-IP) assay validating the interaction in lysates from the medial shell of the nucleus accumbens following CART peptide microinjection.

**Blots raw data**


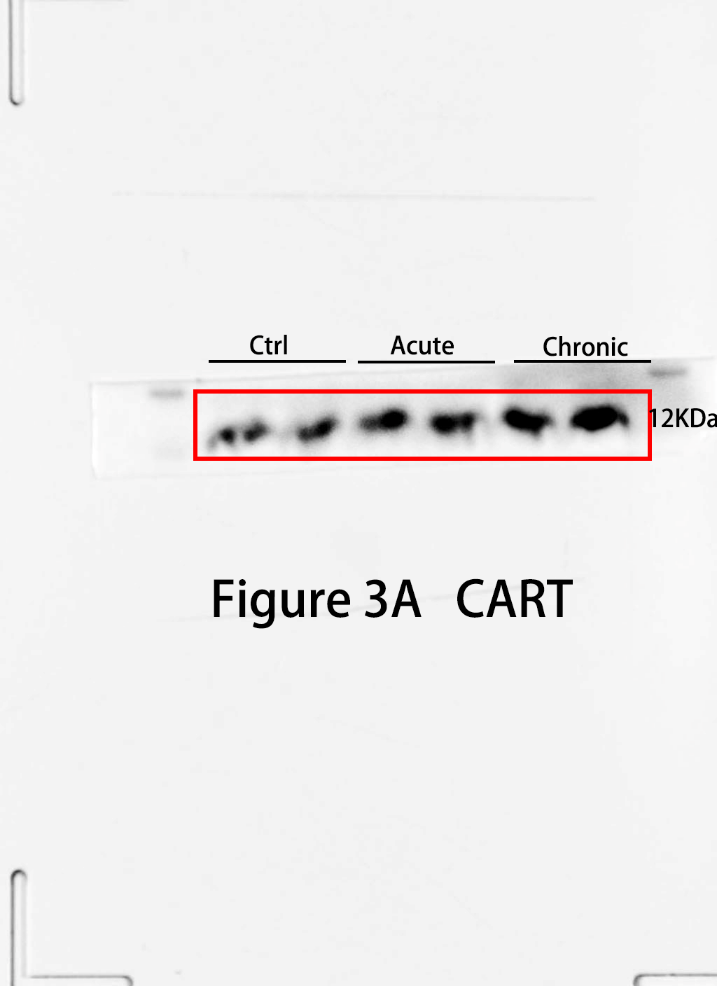


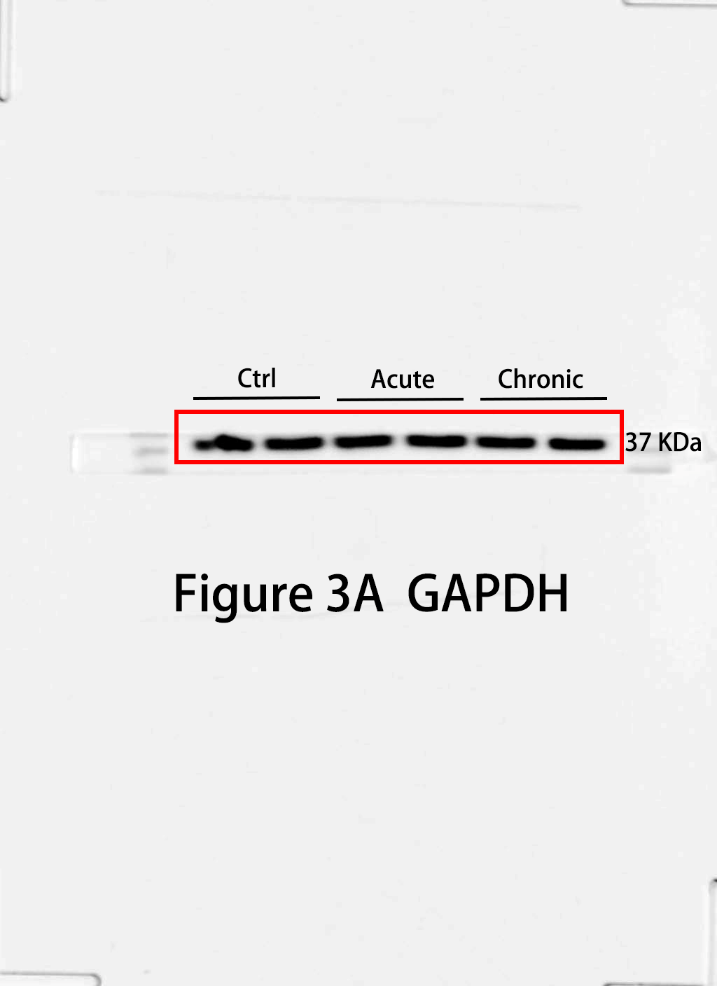

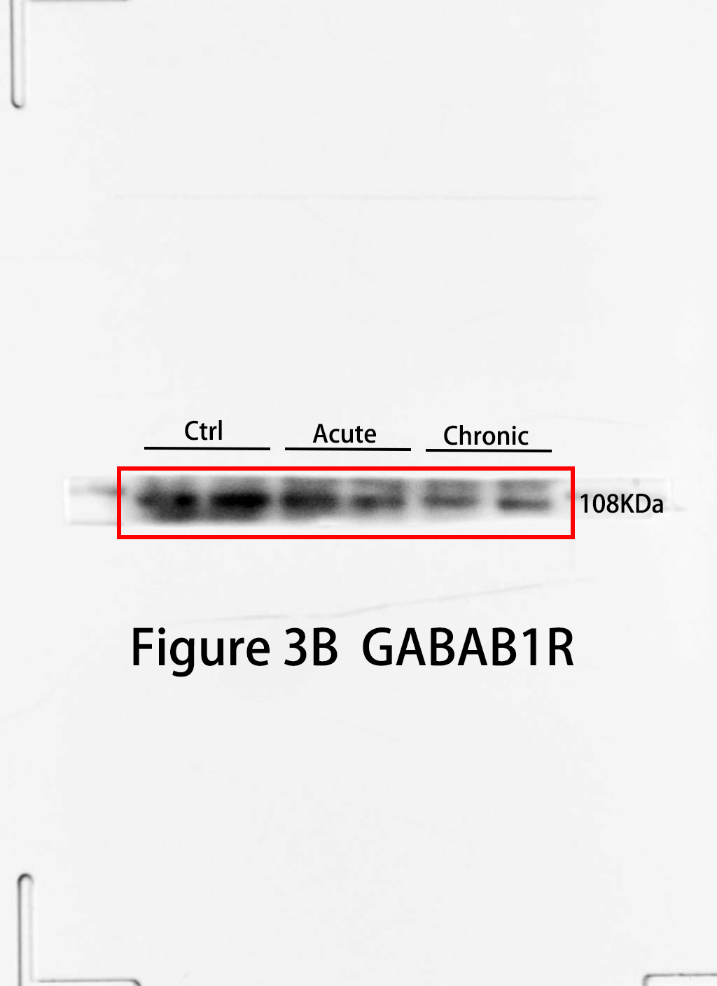


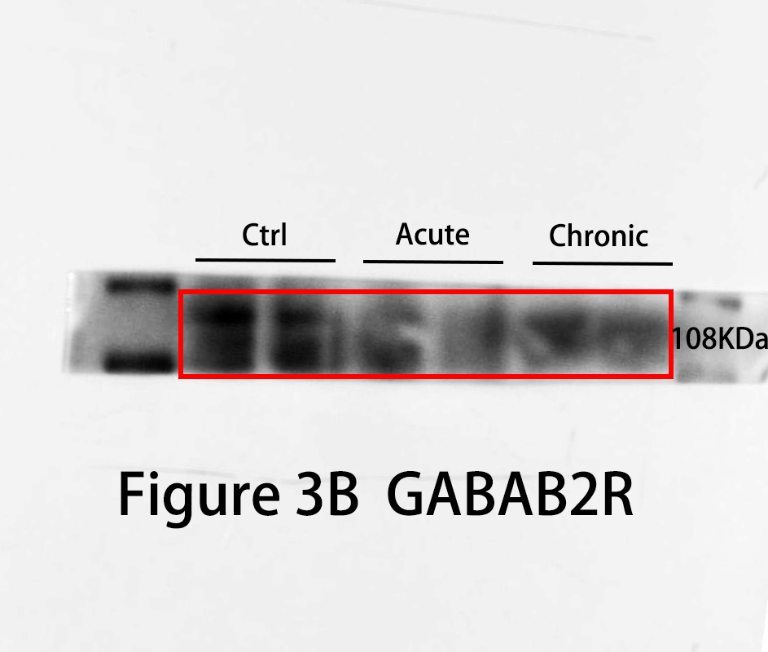


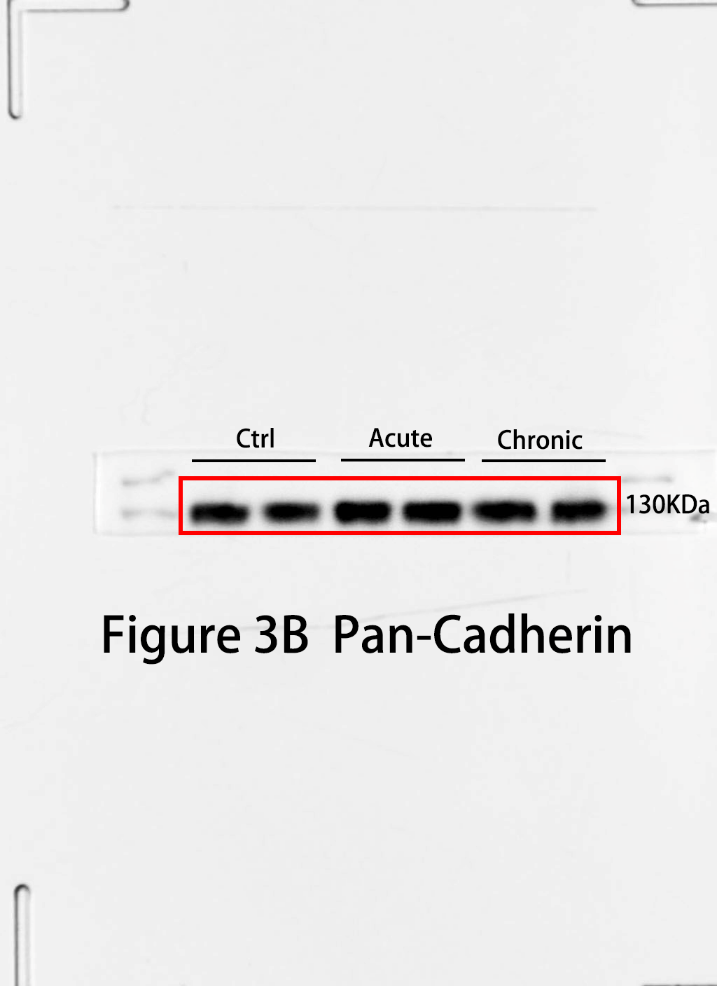


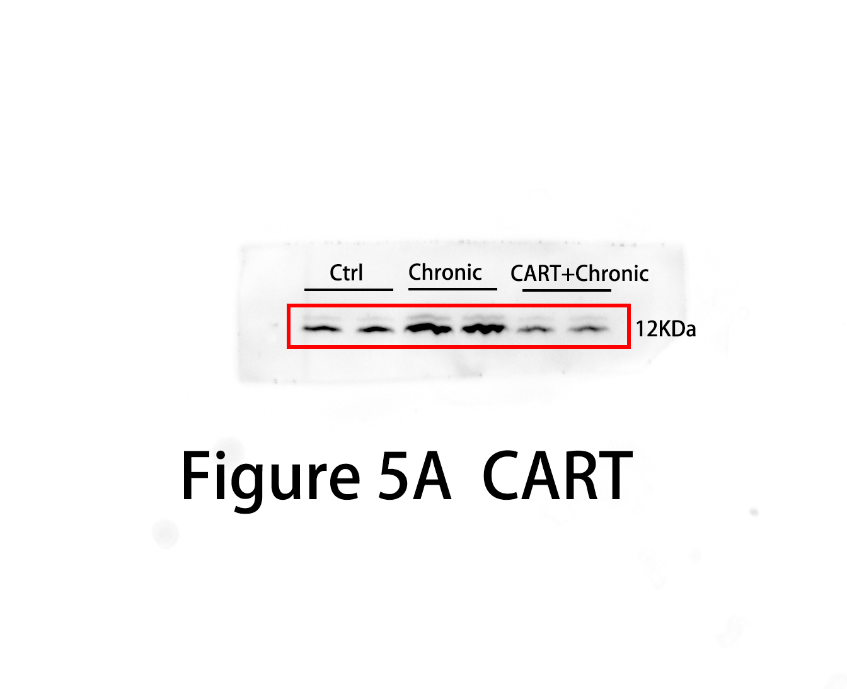


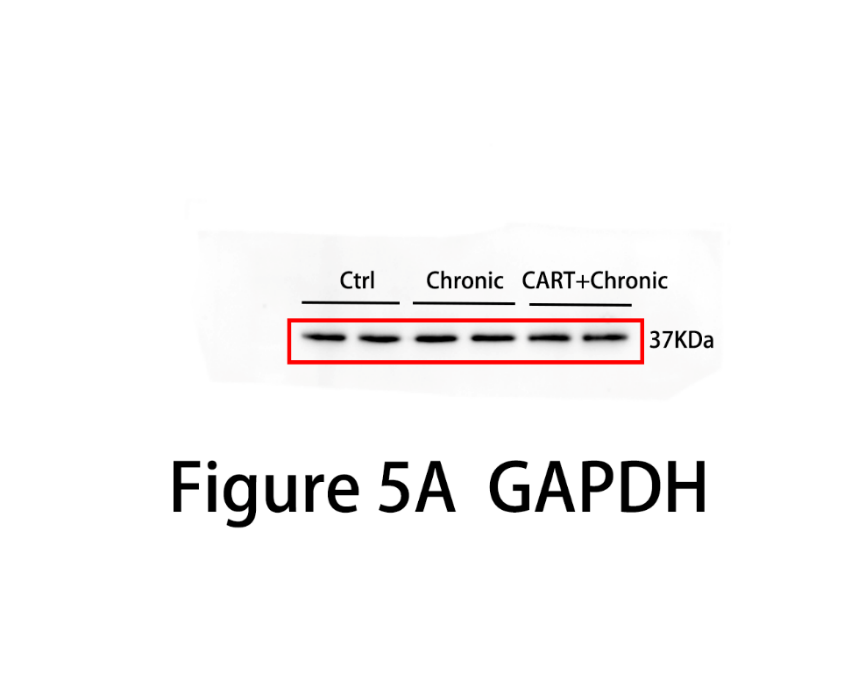


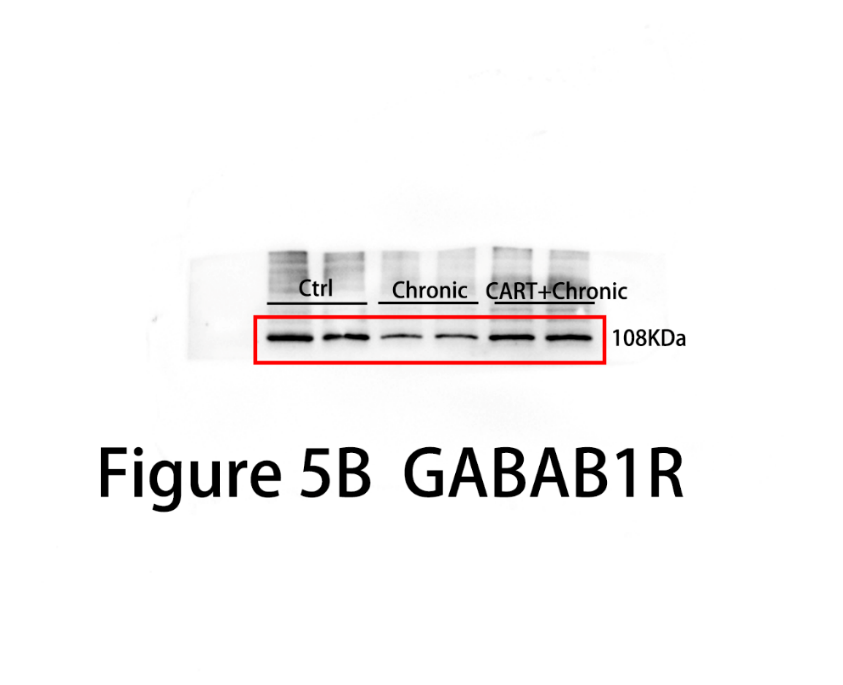


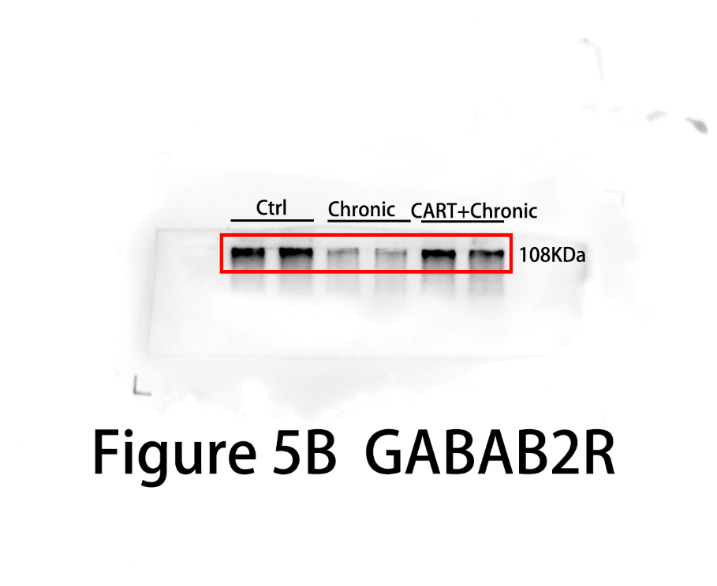


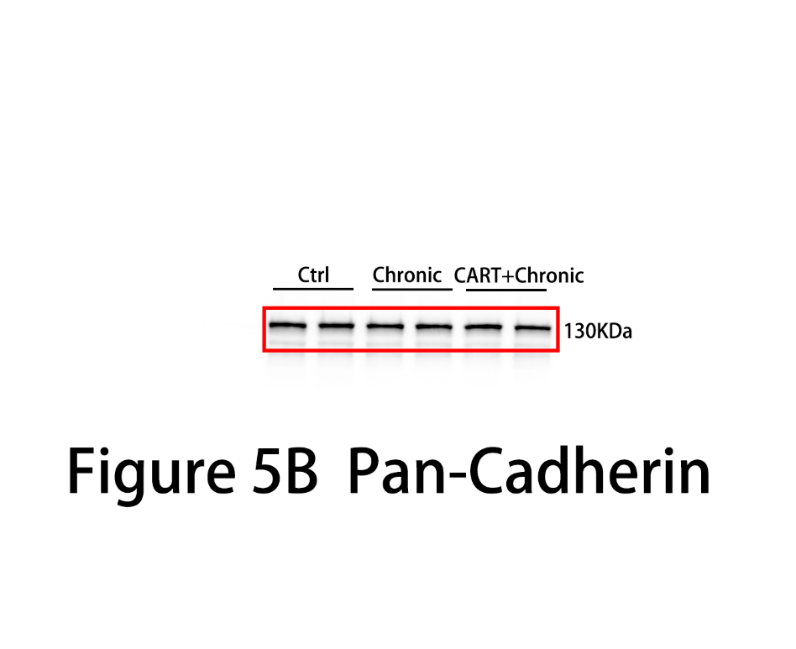


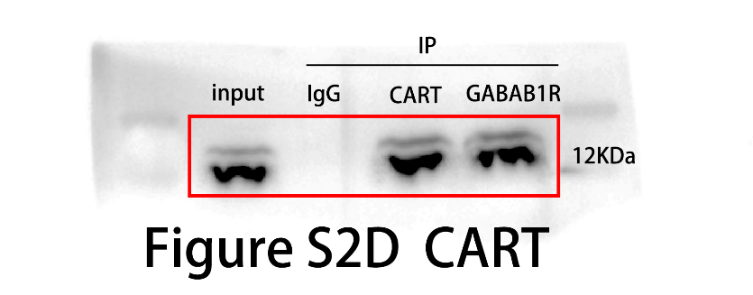


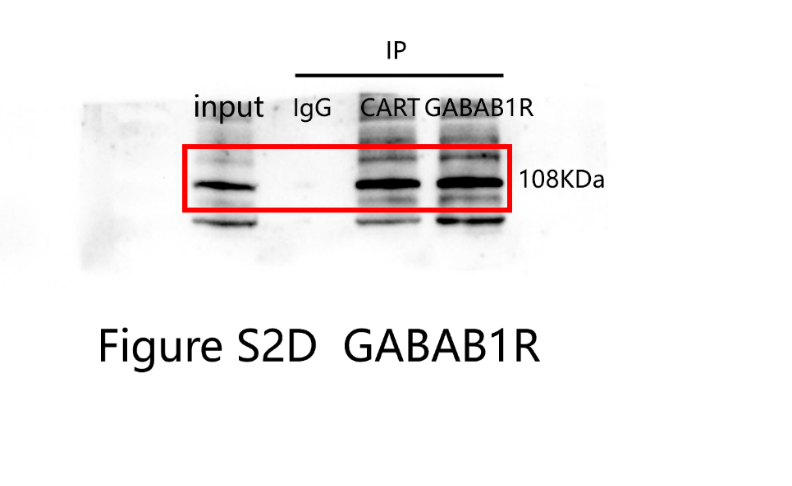

Supplement: Supplementary file 1 — Supplementary Material 1 [file 41598_2026_46389_MOESM1_ESM.docx]
